# Hydrogen-Bonded Organic Framework Enables Phase-Pure Layered Tin Perovskite Nanowires for Room-Temperature Lasing

**DOI:** 10.1021/jacs.5c14431

**Published:** 2026-01-02

**Authors:** Jeong Hui Kim, Jeffrey Simon, Wenhao Shao, Zhichen Nian, Hanjun Yang, Peigang Chen, Brandon Triplett, Zhixu Li, Pengfei Wu, Yuheng Chen, Henna Farheen, Karthik Pagadala, Kyu Ri Choi, Colton B. Fruhling, Jens Förstner, Alexandra Boltasseva, Brett M. Savoie, Vladimir M. Shalaev, Letian Dou

**Affiliations:** † Davidson School of Chemical Engineering, 1371 Purdue University, West Lafayette, Indiana 47907, United States; ‡ Elmore Family School of Electrical and Computer Engineering, 311308 Purdue University, West Lafayette, Indiana 47907, United States; § Birck Nanotechnology Center, Purdue University, West Lafayette, Indiana 47907, United States; ∥ Department of Chemistry, University of Georgia, Athens, Georgia 30602, United States; ⊥ Department of Chemistry, 311308 Purdue University, West Lafayette, Indiana 47907, United States; # Theoretical Electrical Engineering, Institute for Photonic Quantum Systems (PhoQs), Paderborn University, Paderborn 33098, Germany; ∇ Research Institute for Nanoscale Science & Technology, Chungbuk National University, Cheongju, Chungbuk 28644, Republic of Korea; ○ Department of Chemical and Biomolecular Engineering, 6111 University of Notre Dame, Notre Dame, Indiana 46556, United States; ◆ Department of Chemistry, Emory University, Atlanta, Georgia 30322, United States

## Abstract

Room-temperature lasing is a key milestone in the development of miniaturized optoelectronic and photonic devices. We present a simple approach to synthesize phase-pure quasi-2D layered tin perovskite nanowires with varying quantum well thicknesses (*n* = 1 to 4). By incorporating a new organic spacer capable of forming a hydrogen-bonded organic framework, this method promoted anisotropic crystal growth and enhanced lattice rigidity. Furthermore, introducing molecular intercalants enabled controlled crystallization into well-defined nanowires that function as Fabry–Pérot cavities. Cavities made from *n* = 2 to 4 perovskites support efficient and robust near-infrared, room-temperature optically pumped lasing with the threshold as low as 75.8 μJ/cm^2^, cavity quality factor over 3000, and negligible degradation over 10^6^ pulses. A cleaved coupled nanolaser was fabricated as a proof-of-concept device for photonic applications.

## Graphical abstract

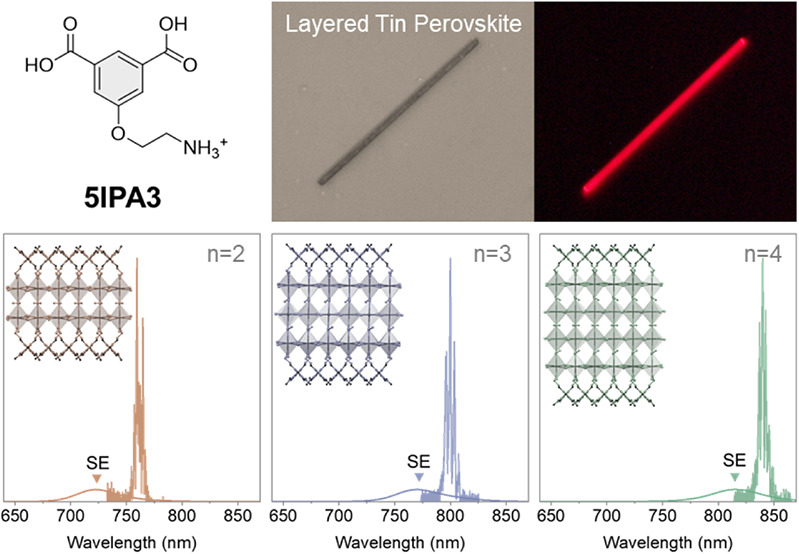

## Introduction

Room-temperature (RT) lasing necessitates efficient gain materials and high-quality cavity resonators. An effective cavity for nanoscale lasers for use in future integrated photonic systems requires careful control over the cavity geometry, for example, through precisely designed crystallization processes. For instance, vertically structured Group III–V semiconductor laser devices used in telecommunications are manufactured with atomically precise epitaxial growth^1,2^ to construct cavities like distributed Bragg reflectors (DBRs) or cleaved Fabry–Pérot (FP) facets.^3^ However, decreasing the size to create a nanolaser is a demanding task: achieving comparable cavity performance at smaller scales requires advanced nanofabrication techniques, including high-resolution lithography and etching, which are often costly and time-intensive.^4^ These limitations drive interest for exploring the capability of low-cost fabrication of new gain materials.^5–7^

Layered metal halide perovskites, also known as two-dimensional (2D) perovskites, have recently emerged as promising solution-processable gain materials^8,9^ with wavelength tunability from ultraviolet (UV), through the visible, to the near-infrared (NIR) spectra.^10^ With their characteristic alternating organic and inorganic lattices resembling quantum wells, these materials synergize a wide molecular design space and encompass superior electronic and photonic properties from the inorganic component.^11,12^ Further, the organic lattices shield the inorganic portions against ion migration, water, and oxygen, which cause chemical instability in bulk 3D halide perovskites.^13^

Notably, cavity control in 2D perovskites could be achieved via bottom-up lattice design, in contrast to the costly top-down nanofabrication required for their III–V counterparts. We recently devised a molecular templating method to control the anisotropy within organic lattices, resulting in scalable fabrication of 1D nanowires from 2D perovskites acting as inherent FP resonators.^14^ Due to the compositional tunability, gain optimization could be subsequently achieved by selecting the tin iodide inorganic lattice instead of lead-based lattices^8,15^ and by increasing the number of inorganic layers in the unit cell (i.e., *n* number) beyond 1 to form quasi-2D phases.^16,17^ Both strategies efficiently reduce exciton–phonon interactions, which are detrimental to optical gain.^18^

However, nanocrystals of high-*n* quasi-2D perovskites commonly contain multiple phases with different *n* numbers that allow for interdomain energy transfer, leading to nonradiative losses. Therefore, phase purity is crucial for effective lasing. While top-down exfoliation helps to obtain phase-pure layered perovskite flakes by breaking at phase boundaries,^19,20^ the direct and deterministic synthesis of phase-pure quasi-2D nanowires is more scalable. This remains inherently challenging due to the complex interplay of chemical, thermodynamic, and kinetic factors governing phase-pure quasi-2D nanowire formation energy. Despite the potential of low-cost RT nanolasers based on quasi-2D perovskites, a precise strategy to simultaneously control their cavity morphology and phase purity remains elusive.

In this work, molecular templating methods were introduced to achieve the scalable synthesis and RT optically pumped lasing of phase-pure quasi-2D tin halide perovskite nanowires. A new organic spacer, named 2-(3,5-dicarboxyphenoxy)­ethan-1-aminium (5IPA3), was designed to establish a directional H-bonding organic framework (HOF) to both improve the structural rigidity and promote 1D crystal growth. Controlled crystallization was further achieved by introducing intercalants into the HOF. Layered tin-iodide perovskite nanowires with various quantum well thicknesses (*n* = 1 to 4) were synthesized in a deterministic and scalable fashion with a remarkable phase purity. Utilizing these nanowires, we demonstrated RT lasing in FP modes under femtosecond laser pumping with low thresholds of 92.8, 75.8, and 131.0 μJ/cm^2^ and reasonable optical gains of 350.3, 600.9, and 404.9 cm^–1^ for *n* = 2 to 4 nanowires, respectively, as well as high quality factors (Q) greater than 3000. These lasers exhibited negligible degradation over 10^6^ pulses and were stable for nanofabrication. We demonstrated the creation of cleaved-coupled nanowire structures for mode manipulation, where two FP cavities formed by adjacent nanowire segments were axially coupled. Our study highlights the controlled synthesis of phase-pure layered tin halide perovskite nanowires. Their naturally formed high-quality cavities make them a promising platform for nanoscale electronics and miniaturized photonic devices.

## Results and Discussion

### Molecular Design Strategies

The bottom-up growth of 1D nanowires of 2D perovskites was achieved with carboxylic acid (COOH)-functionalized organic spacers. The 1D growth originates from the directional HOF that drives the anisotropic population of dangling COOH on growth facets and the resulting surface energy modulation in aqueous media.^14^ This inspired tuning the connectivity of the HOF lattice to enhance the structural stability and rigidity of layered perovskites, both of which reduce the nonradiative losses due to lattice vibrations under high-energy excitation.^21^

We hypothesized that HOF created by spacers with a phthalic acid–based backbone (i.e., isophthalic, terephthalic, or phthalic acid) would enhance the structural robustness of layered perovskites, compared to spacers made from functionalized benzoic acid, like the benchmarked BrCA3. We previously investigated the perovskite nanowire growth using a spacer with the terephthalic acid backbone, 2-(2,5-dicarboxyphenoxy)­ethan-1-aminium (TPA3).^14^ Unfortunately, the strong strain in TPA3-mediated HOF was transferred to the large inorganic octahedral distortion, which diminished the photoluminescence (PL).

To overcome these limitations, we selected the isophthalic acid backbone and, hence, the new spacer 5IPA3 (Figure 1a). This design enabled the formation of an expanded HOF that still extended in one dimension, yet now bridged the original pseudo-van der Waals gap in TPA3-mediated HOF (Figure 1b and Figure S2a-b). Face indexing of the (5IPA3)_2_PbBr_4_ bulk crystal identified the side facets as (010), (0-10), (011), and (0-11) (Figure S3a). The crystal was observed to predominantly grow along the [100] direction. This anisotropic growth behavior is consistent with our previous findings,^14^ suggesting that surface termination and morphology are governed by the presence of dangling carboxylic acid moieties (Figure S3b), which stabilize low-energy crystal surfaces.

**Figure 1:**
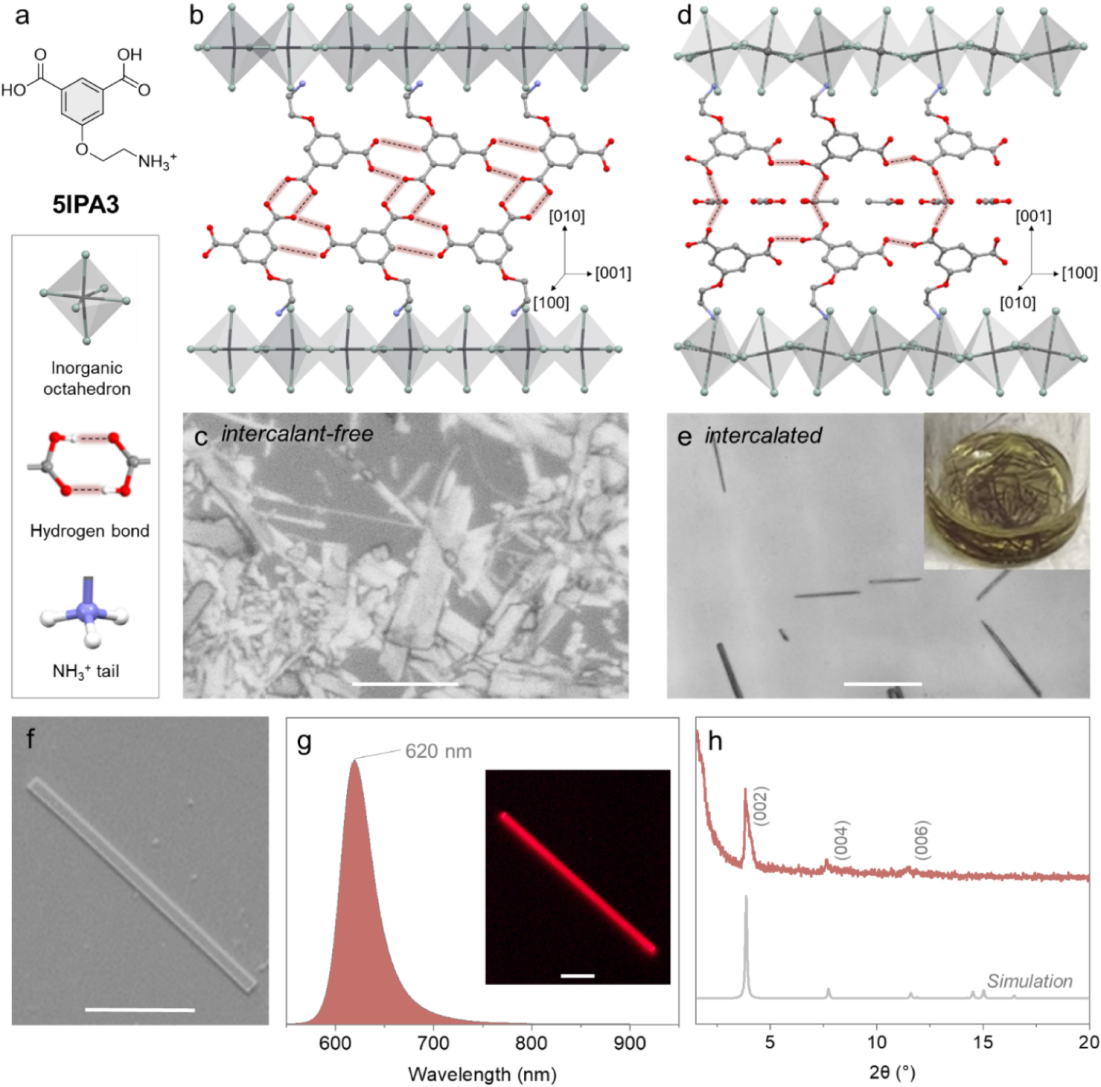
A comparison of the morphology and crystal structure between intercalant-free and intercalated nanowires. (a) Chemical structure of the 5IPA3 organic spacer. (b) The crystal structure of (5IPA3)_2_PbBr_4_ viewed along the [100] direction. Hydrogen atoms were omitted for clarity. (c) Morphology of intercalant-free (5IPA3)_2_SnI_4_ nanowires under a bright-field optical microscope (scale bar: 10 μm). (d) Intercalated (5IPA3)_2_SnI_4_ crystal structure along the [010] direction. Hydrogen atoms were omitted for clarity. (e) Morphology of intercalated (5IPA3)_2_SnI_4_ nanowires under a bright-field optical microscope (scale bar: 500 μm) and (inset) nanowires suspended in the growth media. (f) SEM image (scale bar: 10 μm) of the intercalated (5IPA3)_2_SnI_4_ nanowire. (g) PL spectrum, (inset) PL image (scale bar: 10 μm), and (h) PXRD data from a large population of intercalated crystals.

Notably, we reconfirmed that the TPA3-based crystal exhibited no detectable emission, whereas the 5IPA3-based counterpart showed clearly observable emissive behavior in PL images (Figure S4a-b), indicating a reduction in excitonic quenching. This observation reflects a subtle lattice perturbation that yielded substantial structural–property repercussions. The octahedral distortion negatively affects the excitonic nature of perovskites^22^ and is characterized by the normalized root-mean-square of the metal-halide bond distance (Δ) (Figure S4c). The introduction of 5IPA3-based 2D perovskite nanowires in comparison to the TPA3-based counterpart decreased Δ from 6.2 × 10^–4^ to 2.3 × 10^–4^. This decrease suggested relaxation of the internal stress within the HOF lattice, which was subsequently transferred to the inorganic layer.

On the other hand, 5IPA3-based structures indeed presented greater structural rigidity than the TPA3 counterpart as hypothesized, possibly due to an expanded HOF. The structural rigidity was quantified by the equivalent isotropic displacement parameters (*U*_eq_) extracted from the refined crystal structure obtained by single-crystal X-ray diffraction, which qualitatively indicated the degree of thermal vibration in a crystal structure. Between the two spacers—5IPA3 (isophthalic) and TPA3 (terephthalic)—5IPA3-based perovskite exhibited lower thermal vibration across all examined atoms (C, N, O, Br, and Pb), indicating high structural rigidity (Figure S4d). It should be noted that *U*_eq_ can also be influenced by static displacive disorder, inherent atomic motion, low-quality data, and single-crystal X-ray data processing. Hence, precise quantitative comparisons should be approached cautiously. Qualitatively, this enhanced rigidity minimizes lattice vibrations, thereby reducing exciton–phonon interactions^21^ and lowering susceptibility to thermalization under high-energy excitation. Therefore, their superior emission properties and structural rigidity make 5IPA3-based 2D perovskites promising candidates for photonic applications.

### Lattice Intercalation and Morphology Control

We adapted the 5IPA3-mediated HOF to a [SnI_4_]^2–^ lattice, motivated by its lower toxicity^23^ and decreased exciton–phonon coupling.^18^ However, Sn­(II), being stronger Lewis acids than Pb­(II), dramatically accelerated lattice assembly and led to rapid and uncontrollable crystal growth when paired with the 5IPA3 spacer.^24^ This resulted in uncontrollable crystallization and excessive aggregation of (5IPA3)_2_SnI_4_ nanocrystals with underdeveloped facets (Figure 1c).

Controlled crystallization necessitates balanced association and dissociation of the precursors. Our molecular templating strategy utilizes the linearly aligned HOF to modulate the surface energy via dangling H-bond donors and acceptors. These “surface antennas” should have a higher population on the side facets of the nanowire (i.e., planes parallel to the long axis) versus the end to successfully promote 1D growth. Suggested by our previous analysis,^14^ nanowire growth using TPA3 or 5IPA3 would be similarly uncontrollable due to the perfectly linearly aligned directional COOH along the <100> direction (Figure S5a-b), which would leave negligible H bond antennas at the nanowire end facets, leaving them unprotected during crystal growth.

Therefore, to effectively regulate the crystallization dynamics, we initially introduced acetic acid as a cosolvent in the conventional mineral acid media (Figure 1d). Yet, the protic acetic acid interacted strongly with 5IPA3 via H-bonding and thus unexpectedly got incorporated into the original HOF. This intercalation led to a 2D secondary lattice embedded in the 5IPA3 network, which consisted of interconnected acetic acid and water molecules and was stabilized by H-bonds with 5IPA3 (Figure S6a). As a result, the *d*-spacing increased from 44.4 to 46.5 Å.

Additionally, the introduction of the intercalant broke the original parallel alignment of 5IPA3 along the [001] axis and induced alternating rotation, leading to the cross-alignment of 5IPA3 molecules along the current [100] axis with an ∼90° angle between their aromatic basal planes (Figure S6b-f). This structural modification disrupted the original COOH dimer network. Thus, the directional COOH moieties were no longer perfectly aligned with the <100> direction, but now at an angle between the current [100] and [010] axes, resulting in an alternative 5IPA3-mediated HOF that extends along the [010] direction (Figure S7a-c), along with a secondary intercalation lattice that extends in 2D (Figure S7d-e).

The new HOF with mixed dimensions would lead to nondiminishing H-bond antennas on all growth facets, which thus completed the regulation of crystallization. As a result, well-defined layered tin perovskite nanowires were obtained (Figure 1e-f), exhibiting PL centered at 620 nm (Figure 1g) with well-defined excitonic features and high morphological purity, as confirmed by both bright field images (Figure 1e) and powder X-ray diffraction (PXRD) across a large population of nanowires (Figure 1h). Furthermore, owing to the rigid HOF structure, (5IPA3)_2_SnI_4_ nanowires show improved environmental stability compared to (PEA)_2_SnI_4_ nanoflake crystals (Figure S8).

To quantitatively assess the effect of intercalation, the binding energies per unit area were calculated for both the intercalant-free and intercalated (5IPA3)_2_SnI_4_ crystal structures (Methods). The calculation results suggested a lower in-plane binding energy in the intercalated structure than in the intercalant-free counterpart (1.04 vs 1.32 kcal/mol·Å^2^) (Figure S9). A lower binding energy on a given surface indicates a less favorable binding site and therefore suggested that intercalation would slow down the crystal association in plane. Overall, this intercalant engineering approach enabled the growth of crystals with distinct end facets, providing an intrinsic optical cavity favorable for light confinement and lasing.

### Phase-Pure Quasi-2D Tin Perovskite Nanowires

The molecular templating strategy described above was then employed for quasi-2D perovskites. The deterministic synthesis of quasi-2D perovskite nanocrystals relies on phase purity to eliminate the need for particle screening or postmodification, such as exfoliation. In general, the phase purity challenge drastically escalates with increasing *n* number due to the complex interplay of chemical, thermodynamic, and kinetic factors that govern their formation energies.^25^ These challenges are evident in both lead- and tin-based systems, with tin iodide candidates being particularly difficult to handle due to the high susceptibility of Sn­(II) to oxidation and the resulting *V*_Sn_ defect during synthesis.^26^

By systematically optimizing precursor stoichiometry, we achieved high phase purity for (5IPA3)_2_MA_*n* – 1_Sn_*n*_I_3*n* + 1_ (*n* = 2 to 4) quasi-2D perovskites, where MA is methylammonium. Notably, the use of 5IPA3 as the spacer cation consistently suppressed phase intergrowth and promoted the selective formation of distinct *n* number phases. We hypothesize that 5IPA3 modifies the potential energy surface during crystallization, effectively deepening and separating the energetic minima corresponding to each targeted phase. However, the mechanism remains under investigation.

As shown in Figure 2a–c, scanning electron microscopy (SEM) confirmed the cavity quality of these nanowires with well-defined edges. Additionally, the measured population-level dimensions of the nanowires (Figure S10-S13) demonstrate elongation along the length in comparison to the width and height. Accordingly, the mean aspect ratios (length/width) are 33.0, 29.7, 42.6, and 28.5 for *n* = 1, 2, 3, and 4 nanowires, respectively. Consistent presence of an intercalation layer and similar HOF lattices were observed across the crystal structures of *n* = 2 to 4 phases (Figure 2a-c insets). It is noteworthy that the *n* = 2 crystal structure did not exhibit acetic acid and water molecules in the intercalation layer due to the application of the SQUEEZE algorithm to account for a severely disordered structure.^27^ However, based on structural similarities with the *n* = 1, 3, and 4 crystals as well as the presence of elements in the intercalation layer that have H-bonding distances to carboxylic acid groups, it is plausible to speculate a similar intercalation lattice in the *n* = 2 phase. This interpretation was further supported by the linear correlation (*R* ^2^ ≈ 0.998) between the experimentally measured *d*-spacing of each phase and its *n* number (Figure S14).

**Figure 2:**
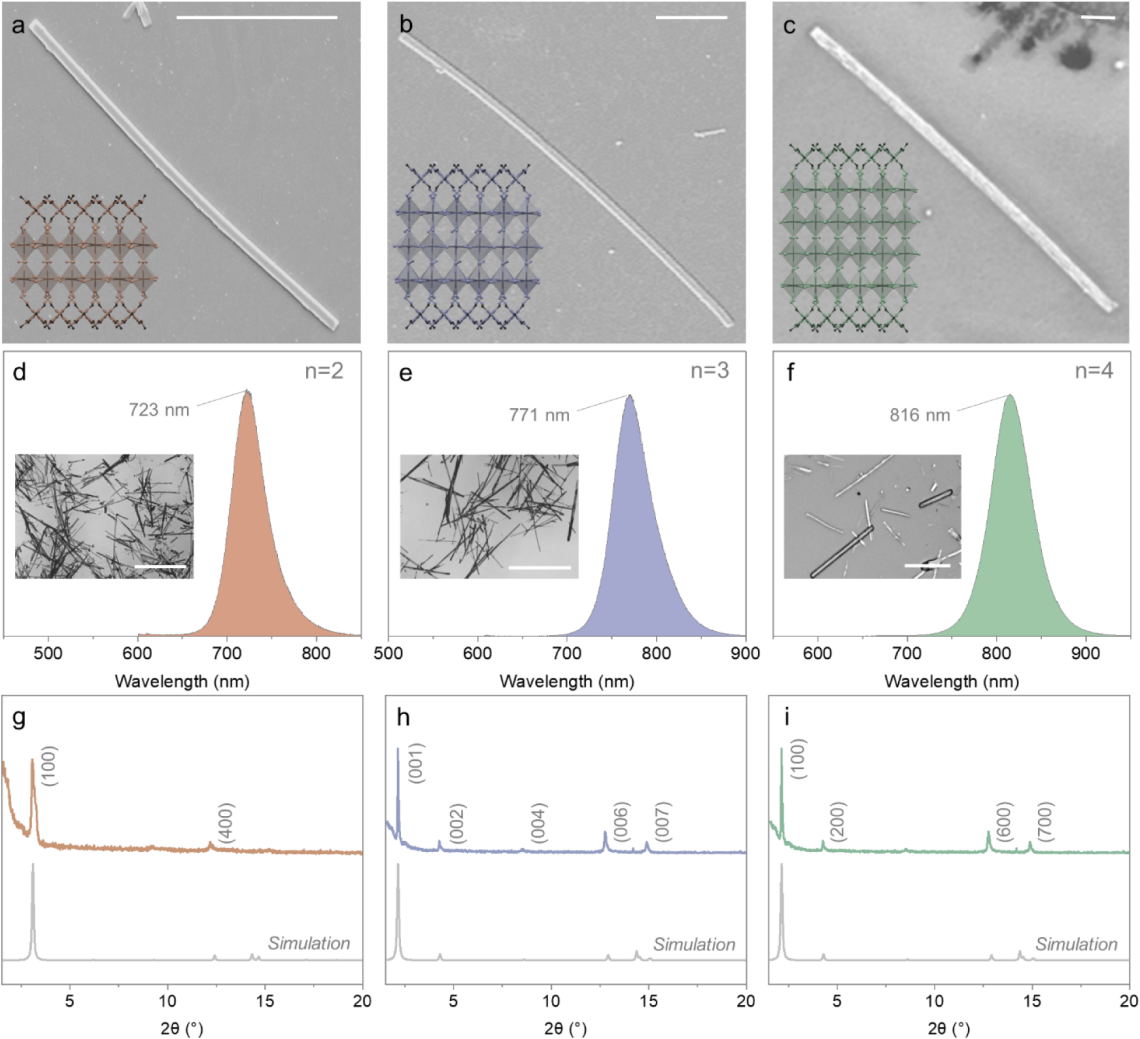
Morphological, optical, and structural characterization of pure-phase quasi-2D (5IPA3)_2_(MA)_*n* – 1_Sn_*n*_I_3*n* + 1_ (*n* = 2, 3, and 4) perovskite nanowires. SEM images of (a) *n* = 2, (b) *n* = 3, and (c) *n* = 4 intercalated perovskite nanowires (scale bars: 10 μm) with insets of the side view of the crystal structures. PL spectra of (d) *n* = 2, (e) *n* = 3, and (f) *n* = 4 intercalated perovskite nanowires. The insets are images of the nanowires on glass substrates (d, e; scale bars: 1 mm) or Si/SiO_2_ wafer (f) (scale bar: 10 μm). PXRD of (g) *n* = 2, (h) *n* = 3, and (i) *n* = 4 intercalated perovskite nanowires. Stacking direction is along c for *n* = 3 and along a for *n* = 2 and *n* = 4.

The excellent phase purity of *n* = 2 to 4 crystals was validated through ensemble-averaged PL (Figure 2d–f) and PXRD (Figure 2g–i) where a large population of crystals was randomly selected and investigated (Figure 2d–f insets). Characteristic excitonic PL at 723, 771, and 816 nm for *n* = 2 to 4 nanowires, respectively, was observed without impurity or shoulder peaks. PXRD spectra revealed distinct and sharp diffraction peaks for (100) at 3.1°, (001) at 2.5°, and (100) at 2.1° for *n* = 2 to 4, respectively. This consistency highlights the exceptional phase purity and reproducibility, demonstrating its effectiveness in producing high-quality, phase-pure quasi-2D perovskite structures.

### Room-Temperature Lasing

RT lasing was investigated with (5IPA3)_2_MA_*n* – 1_Sn_*n*_I_3*n* + 1_ (*n* = 1 to 4) nanowires deposited on Si/SiO_2_ substrates and measured under argon ambient conditions. Among the examined samples, the *n* = 1 composition did not exhibit RT lasing even under a high pump fluence of 751 μJ/cm^2^ (Figure S15). Alternatively, experiments with *n* = 2 to 4 nanowires showed lasing at wavelengths centered around 762.5, 801.0, and 865.0 nm for *n* = 2 to 4, respectively, indicating efficient wavelength tunability in the NIR (Figure 3a). Lasing evidence included emission lifetime reduction (Figure 3b), clear threshold behavior (Figure 3c and Figure S16-S18), and peak narrowing (Figure 3d). Reflecting the dominance of stimulated emission at pump powers above the lasing threshold (*P*_th_), at least a 4-fold decrease in average emission lifetime was measured via time-resolved PL (TRPL) and fit with a biexponential decay (Table S2). The sharp PL peaks emerged when the samples were pumped above the lasing threshold power. The lowest lasing thresholds were 92.8, 75.8, and 131.0 μJ/cm^2^, and the mean lasing thresholds were 329.1, 163.3, and 340.3 μJ/cm^2^ across a respective population of 24, 27, and 7 *n* = 2 to 4 nanowires (Figure S19).

**Figure 3:**
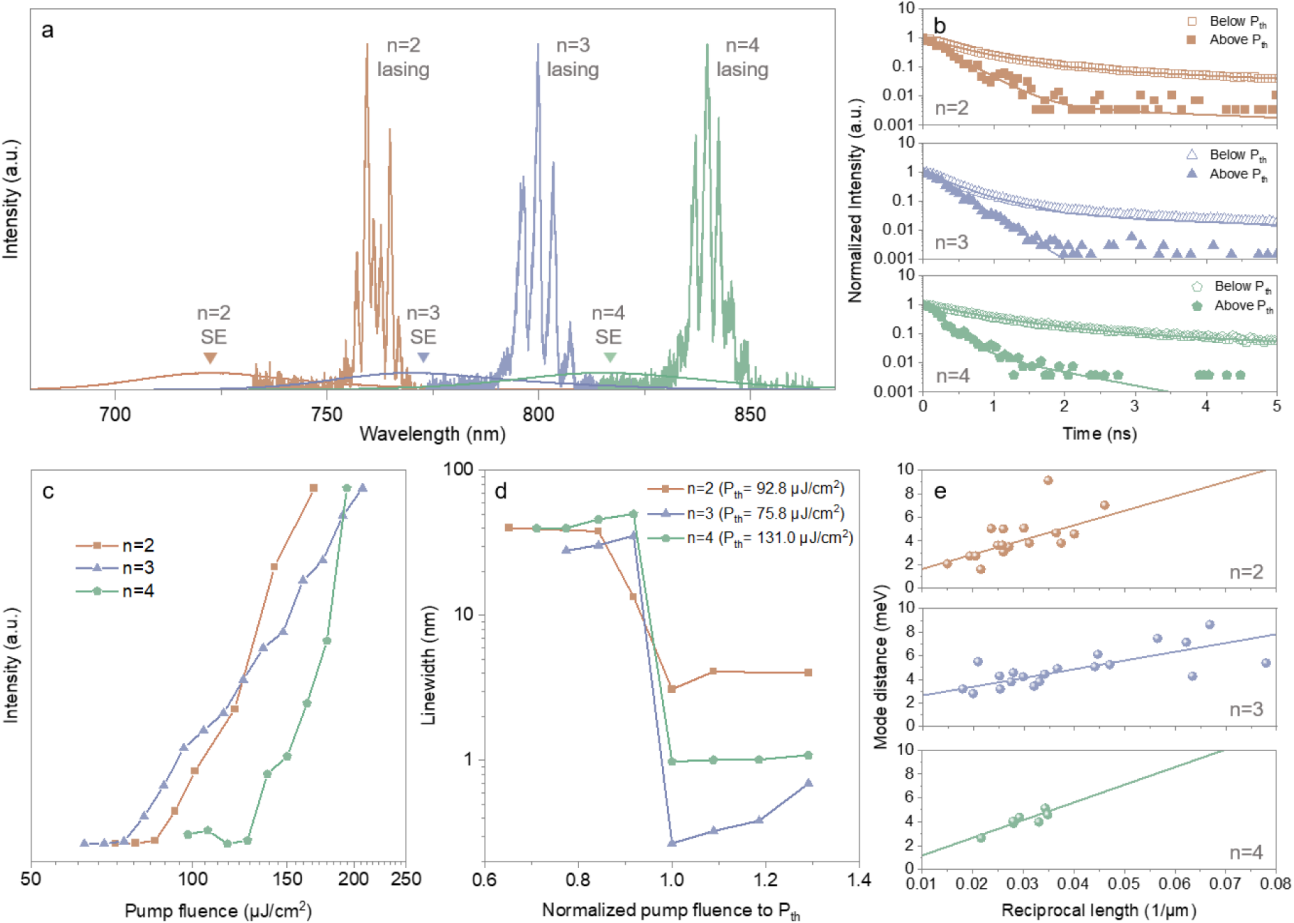
RT lasing characteristics of 2D perovskite nanowires. (a) Spontaneous emission (SE) and lasing spectra (1200 gr/mm grating) of (5IPA3)_2_(MA)­Sn_2_I_7_ (*n* = 2), (5IPA3)_2_(MA)_2_Sn_3_I_10_ (*n* = 3), and (5IPA3)_2_(MA)_3_Sn_4_I_13_ (*n* = 4) perovskite nanowires. (b) TRPL curves measured at fluences below (3.98 nJ/cm^2^) and above the lasing threshold (878.42 μJ/cm^2^). (c) Input–output characteristics showing the lasing threshold of the lowest threshold *n* = 2 to 4 perovskite nanowires. (d) Emission full width at half-maximum (fwhm) across the lasing threshold. Pump fluences were normalized to *P*_th_. A distinct decrease in line width marks the transition from spontaneous to stimulated emission. (e) The mode spacing of lasing peaks in lasing PL data plotted with respect to inverse cavity length.

Nanowire forms a FP cavity where light that is confined to waveguide modes travels across the length of the wire, and reflects at the edge facets.^28^ Waveguide modes originate from the confinement of light to the small cross-section cavity dimensions on the order of the wavelength of light and are corroborated through optical modeling (see Note S1). The spacing of the adjacent FP resonance lasing modes is expected to be inversely proportional to the length and effective refractive index of the cavity,^29^ which was confirmed in our experiments (Figure 3e).

The cavity quality factor reflects the morphological control of the nanowires. The nanowire cavities have high *Q* factors, with the mean reaching over 1500 and the maximum around or greater than 3000 across all *n* numbers (Figure S20). To further evaluate their lasing characteristics, we compared key parameters, including lasing threshold and Q factor, among various 2D or quasi-2D perovskite single-crystal lasers (Table S3). Samples that did not create well-defined cavities exhibited significantly higher lasing thresholds (Figure S21-S22), which further exemplifies the critical role of the FP nanowire cavity in achieving efficient lasing. High *Q* correlates with high reflectivity of light at wire’s end facets and a longer wire length *L*.^28^ The experimental observation of a linear dependence of *Q* on cavity length *L* (*n* = 3; Figure S23) suggested that facet quality was preserved in a wide range of crystals with various dimensions and hence an excellent morphological control.

A representative *n* = 3 nanowire exhibited strongly linearly polarized lasing emission with a high degree of polarization of ∼93% (Figure S24) and a polarization direction perpendicular to the long axis of the nanowire and parallel to the plane of the substrate, consistent with previous reports.^30^ This polarization result suggested that the fundamental lasing mode in our nanowire was thus predominantly transverse electric, which was shown to be supported by the nanowire cavity (see Note S1).

### Multimode Dynamics

The well-defined cavities enabled multimode lasing dynamics, as evidenced by the multiple lasing peaks. The power-dependent spectral evolution of the lasing modes was analyzed by fitting the peak positions and amplitudes (Figure S25). At higher powers, more lasing peaks appeared on the red side of the spectrum, and power saturation occurred on the blue side, which resulted in an apparent bathochromic spectral shift (Figure S25a-b). This saturation dynamic may result from self-absorption of higher-energy modes during light propagation or a mode competition where lower energy modes experienced lower losses and consumed more of the material’s gain. The absence of a dominant mode could also have stemmed from inhomogeneous broadening or spatially varying gain saturation.^31,32^ Additionally, individual lasing modes exhibited a progressive blue shift with increasing pump fluence (Figure S25c), attributed to nonlinear refractive index changes within the nanowire cavity at higher pump power.

Multiple lasing peaks arise from the interplay between transverse waveguide modes and FP resonances and are further affected by nonlinear optical processes. We observed a small free spectral range (FSR) in lasing spectra inconsistent with a single set of FP resonances. After considering two sets of FP resonances, the FSR extracted effective refractive indices (*n*_eff_) of 2.37, 2.38, and 2.11 for *n* = 2 to 4 nanowires, respectively, align with reported values.^8,33^ Note S1 describes the analysis and calculations. Engineering the multiple FP resonances and additionally the correlated nonlinear effects^34^ in this nanolasing system could lead to novel photonic device designs.

### Exciton–Phonon Coupling and Effective Gain

The observed lowest *P*_th_ in *n* = 3 nanowires versus *n* = 2 and 4 was investigated further by evaluating their inherent material properties and overall gain properties. In the lasing regime, a major advantage of higher *n* number quasi-2D layered perovskites over the *n* = 1 counterpart is their suppressed exciton–phonon interactions. Temperature-dependent PL measurements of *n* = 1 to 4 nanowires (Figure S26) revealed the respective phonon coupling constants (Γ_LO_) of 311.1, 173.6, 129.8, and 110.6 meV (Figure S27 and Table S4). The monotonic decrease of coupling strength with increasing *n* number along with a major reduction from *n* = 1 to 2 was primarily attributed to the increased structural rigidity of the inorganic layers. The higher rigidity lowers the lattice phonon energy by providing a firmer lattice environment and weakening the interactions between excitonic wave functions and lattice vibrations.^35^ This decreasing exciton–phonon coupling strength has significant implications for the optoelectronic and lasing regimes. In layered perovskites with higher *n* number, the weaker exciton–phonon interaction results in lower nonradiative recombination rates, reduced phonon-assisted Auger recombination, and minimized energy losses.^36^

On the other hand, the effective optical gain (*G*_eff_), which denotes the gain minus losses, can be used to evaluate the material’s effectiveness as a lasing media. *G*_eff_ was evaluated using the variable stripe length (VSL) method.^37^ The maximum *G*_eff_ was highest in *n* = 3 nanowires (600.9 cm^–1^), compared to *n* = 2 (350.3 cm^–1^) and *n* = 4 (404.9 cm^–1^). However, the mean, *G̅*_eff_, was 249.8, 246.4, and 232.8 for *n* = 2 to 4 nanowire samples, respectively (Figure S28), which indicated a high experimental variability.

As the cavity quality factor represents morphological control, the effective optical gain is the difference between the inherent material gain and nonradiative losses via phonon coupling, impurities, internal energy transfer, and other factors. These could be further investigated through analysis of the power-dependent TRPL measurements.^38^ While *Q* and *G*_eff_ both contribute to the observed lasing threshold, the similar values observed in perovskite nanowires with various *n* numbers suggested that other factors are contributing to the observed lowest lasing threshold in the *n* = 3 devices. However, due to the limited number of samples measured in the gain experiments, it is difficult to draw a definitive conclusion regarding the origin of the threshold differences.

### Stability and Robustness for Nanofabrication

The excellent morphological and purity control determines the robustness and operational stability of our quasi-2D tin perovskite nanowire lasers under pulsed excitation. Negligible degradation over 10^5^–10^6^ pulses was observed (Figure 4a and Figure S29–30). This robust performance indicated the potential of these materials for practical and stable applications in integrated photonics.

**Figure 4:**
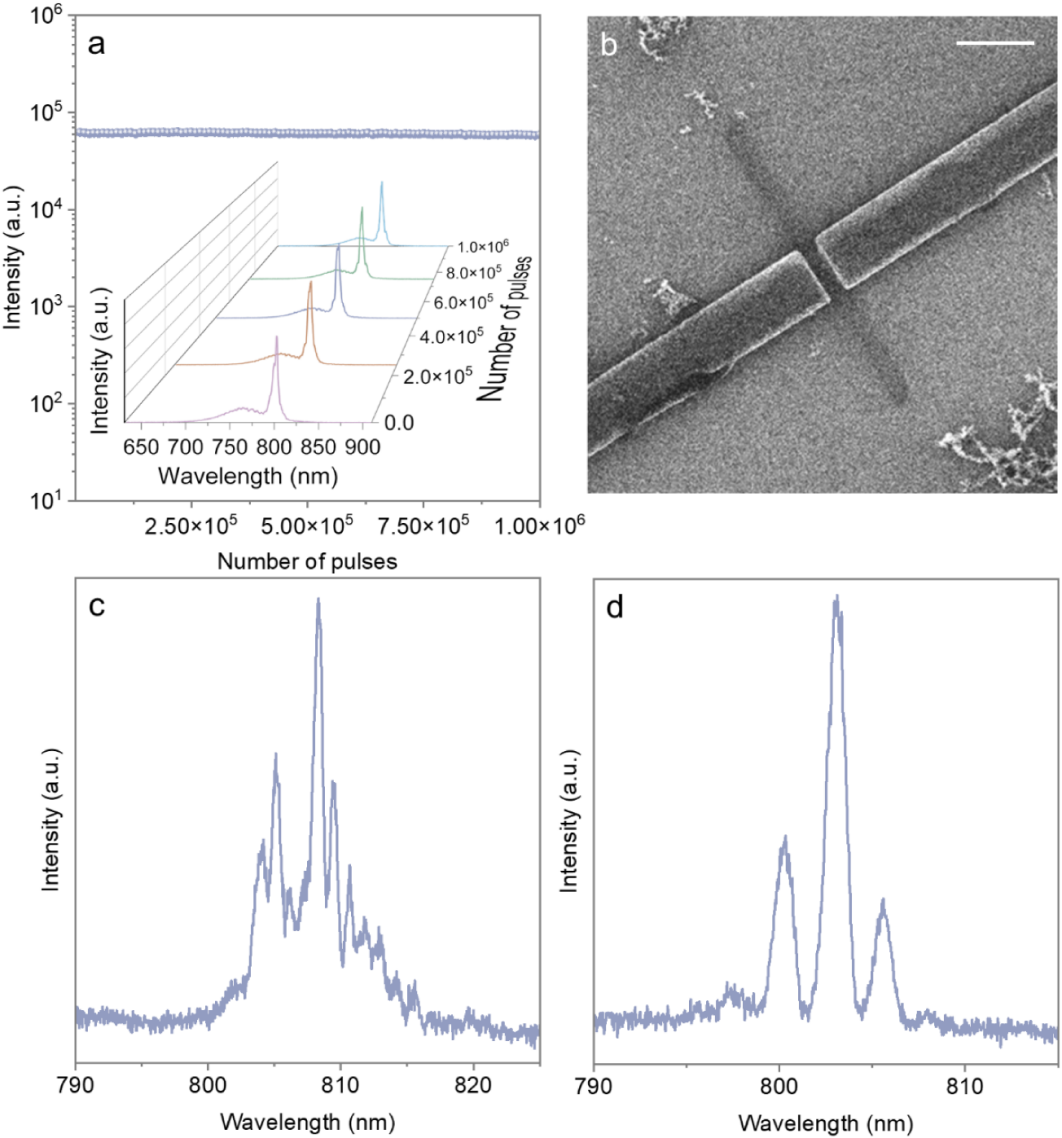
Coupled perovskite nanowire lasing. (a) Stability and evolution of lasing spectra (inset) of the (5IPA3)_2_(MA)_2_Sn_3_I_10_ (*n* = 3) nanowire at RT at the repetition rate of 1 kHz, collected using a 300 gr/mm grating. (b) Helium ion microscopy images showing axially coupled (5IPA3)_2_(MA)_2_Sn_3_I_10_ nanowire on Si/SiO_2_ substrate (scale bar: 2 μm). (c) Output spectrum of the nanowire before cutting. (d) Lasing in 4:3 cleaved-coupled nanowires, which exhibits clean lasing and a reduced number of modes.

To illustrate the potential integration of layered perovskite nanowires into photonic systems, we fabricated a cleaved coupled perovskite nanowire laser structure. The device was realized by precisely cleaving a nanowire on a Si/SiO_2_ substrate using focused ion beam (FIB) milling (Figure 4b and Figure S31). A significant mode reduction was observed (Figure 4c-d), while the lasing threshold remained largely unaffected (Table S5), indicating that the cavity quality and effective gain were preserved. The experimentally measured value of FSR of the unmodified nanowire exhibited agreement with the calculated value based on a previous linear regression analysis in Figure 3e (*n* = 3). After cleaving, the FSR of the two independent wire segments, as calculated from the linear regression model, deviated substantially from the experimentally observed values, providing evidence of optical mode coupling across the entire cleaved device. This phenomenon, consistent with previous findings in the literature,^39^ suggests that evanescent field interactions between the cleaved segments facilitate the formation of coupled cavity modes, effectively altering the expected mode distribution. More importantly, the ability to fabricate such cleaved coupled nanowire cavities without significantly affecting the lasing threshold shows the robustness of the perovskite material and demonstrates precise control over the lasing modes.

## Methods

### Materials

Lead bromide (PbBr_2_, 99.999% trace metals basis), tin iodide (SnI_2_, AnhydroBeads, −10 mesh, 99.99% trace metals basis), hydrobromic acid (HBr, 48 wt % in H_2_O), hydroiodic acid (HI, 57 wt % in H_2_O), hypophosphorous acid (H_3_PO_2_, 50 wt % in H_2_O), and acetic acid (≥99.7%) were obtained from Millipore Sigma. Methylammonium iodide (MAI, >99.99%) was purchased from Greatcell Solar Materials. Deuterated dimethyl sulfoxide (DMSO-d6) and deuterated chloroform (CDCl_3_) were purchased from Millipore Sigma.

### Synthesis of 5IPA3, 2-(3,5-Dicarboxyphenoxy)­ethan-1-aminium Iodide/Bromide

Step (1): Dimethyl 5-hydroxyisophthalate (from Ambeed, 10 mmol, 1.0 equiv), potassium carbonate (K_2_CO_3_, from Millipore Sigma, 20 mmol, 2.0 equiv), cesium carbonate (Cs_2_CO_3_, from Millipore Sigma, 5 mmol, 0.5 equiv), and *tert*-butyl (2-bromoethyl)­carbamate (from Ambeed, 20 mmol, 2.0 equiv) were placed in a round-bottom flask (RBF) with a magnetic stir bar. The flask was sealed with a rubber septum and purged with argon briefly. Anhydrous acetonitrile (MeCN, Millipore Sigma, 50 mL) was then added via a syringe. After extensive purging with Ar, the reaction mixture was stirred and refluxed at 90 °C for 12 h. Upon completion, the reaction mixture was cooled to room temperature.

Water was added to the mixture, and the crude product was extracted with chloroform. The organic layers were combined, dried over magnesium sulfate, and filtered. The residue was subjected to purification by silica gel column chromatography using a hexane/ethyl acetate gradient. Notably, the product and starting material often exhibit similar *R*_*f*_ values, making them difficult to separate by chromatography. The residual starting material present in the product can be effectively removed in a subsequent purification step following conversion of the product to its corresponding ammonium salt.

Step (2): To an RBF equipped with a magnetic stir bar, the Boc-protected amine obtained from the previous step (1 mmol, 1.0 equiv) was added, followed by anhydrous 1,4-dioxane (from Millipore Sigma, 0.2 M). After the compound fully dissolved, 57% aqueous hydroiodic acid (from Millipore Sigma, 10 mmol, 10.0 equiv) was added for iodide salt formation or 48% aqueous hydrobromic acid (from Millipore Sigma, 10 mmol, 10.0 equiv) for bromide salt formation. The RBF and condenser were thoroughly purged with argon, and the mixture was stirred and refluxed at 90 °C for 12 h. Upon completion, the reaction mixture was cooled to room temperature.

Solvents, including residual water from aqueous HBr or HI, were completely removed by rotary evaporation under reduced pressure. The resulting crude residue was then reprecipitated at least three times by dissolving in acetone, followed by the slow addition of diethyl ether to induce precipitation.

### Computational Methods

All calculations, including geometry optimizations and single-point energy evaluations, were performed using CP2K^40^ at the GFN1-xTB level of theory.^41^ The crystal structure of (5IPA3)_2_SnI_4_ synthesized with the intercalant was first geometry-optimized to obtain an idealized reference structure. For geometry optimization, all structures were fully relaxed until the force on atoms was below 0.02 eV/Å and the atomic displacement was less than 5 × 10^–3^ Angstrom.

Since the experimentally resolved structure of nonintercalated (5IPA3)_2_SnI_4_ was not available, it was approximated based on the known structure of nonintercalated (5IPA3)_2_PbBr_4_. The following steps were taken to construct the model:

(1) The (5IPA3)_2_PbBr_4_ structure was first geometry-optimized.
(2) All Pb atoms were replaced with Sn, and all Br atoms were replaced with I.
(3) The resulting structure was further geometry-optimized.

To calculate the in-plane binding energy for both the intercalated and nonintercalated (5IPA3)_2_SnI_4_ structure, (100) surface slabs were generated. The surface slabs were derived from cleaving the crystal along the desired surface and adding a 20 Å vacuum layer perpendicular to the cleaved structures. The schematic illustration for constructing the intercalated (5IPA3)_2_SnI_4_ structure is shown as an example.

### Perovskite Nanowire Bulk Crystal Growth

Bulk crystals for single crystal X-ray diffraction (SC-XRD) were made through cooling the precursor solution down slowly. The precursor solution contains organic spacer, SnI_2_, and MAI with HI and H_3_PO_2_ mixture solvent with acetic acid as an additive. For (TPA3)_2_PbBr_4_ and (5IPA3)_2_PbBr_4_, PbBr_2_ and HBr without intercalants were used instead of the SnI_2_ and HI/H_3_PO_2_ mixture. The detailed amounts of each material are described in Table S1. The weighing process of SnI_2_ was done in a N_2_-filled dry glovebox. The vial containing solids was then transferred to a separate N_2_-filled glovebox for wet synthesis, where solvents were subsequently added. The vial with the mixture was then transferred to a chemical hood in air, sealed with Teflon tapes, and heated carefully with a heat gun to over 100 °C to induce mild refluxing of solvents until materials were completely dissolved and the solution was clear. This process was carried out with great caution in a fume hood with the sash closed. Sonication was occasionally used to assist in the dissolution of materials, but magnetic stirring was avoided. The vials were placed in a Dewar flask containing boiling water, which was stored for 4–5 days until fully cooled to room temperature. The crystals were stored in the mother liquid until ready to be examined for structural solvation.

### Perovskite Nanowire Fast Cooling Growth

Thinner perovskite crystals for several experiments, including PXRD, SEM, and lasing, were grown with fast cooling growth. For the fast-cooling growth, we use the same precursor solution with bulk crystal growth. The entire synthesis was carried out in a N_2_-filled glovebox used for wet synthesis. After heating up the vial containing precursors and solvents in a heating block (VWR) at 120–140 °C (temperature setting on the hot plate—actual temperature in the heating block was not monitored with a sensor) to fully dissolve the materials, it was left undisturbed until cooled down naturally to room temperature inside the heating block and on the hot plate. After being precipitated, the crystals were transferred to polydimethylsiloxane (PDMS), and solvents were absorbed with a piece of filter paper. To completely dry out, the crystals on PDMS were stored under active vacuum for 5 h; then, the particles were transferred to Si or Si/SiO_2_ by stamping.

### Nuclear Magnetic Resonance (NMR)

NMR was acquired with a Bruker Avance III HD 400 MHz spectrometer equipped with a 2-channel Nanobay console and a 5 mm BBFO Z-gradient SmartProbe at room temperature, or Bruker NEO 500 MHz equipped with a Prodigy liquid-nitrogen cooled cryoprobe with BBFO configuration. Spectral analysis was performed using the MestReNova software package.

### Single Crystal X-ray Diffraction

All of the tin perovskite single crystals for investigation were coated with polybutene oil. Single-crystal X-ray diffraction data were collected using a Bruker D8 Quest Single Crystal Diffractometer equipped with a Cu Kα radiation (λ = 1.54178 Å) I-μ-S microsource X-ray tube, a laterally graded multilayer (Goebel) mirror for monochromatization, and a Photon III C14 area detector (10 × 14 cm). The measurements were performed at 150 K using an Oxford Cryostream 800 variable temperature device.

### Powder X-ray Diffraction (PXRD)

PXRD data were collected in focusing mode on a Panalytical Empyrean X-ray diffractometer equipped with Bragg–Brentano HD optics, a sealed tube copper X-ray source (λ = 1.54178 Å), Soller slits on both the incident and receiving optics sides, and a PixCel3D Medipix detector. Data were collected between 1.5° and 50° in 2θ using Panalytical Data Collector software.

### Scanning Electron Microscopy (SEM)

Morphology of the perovskite nanowires was characterized using a scanning electron microscope (Teneo Volumescope FEG) in Everhart–Thornley detector mode, operated at an accelerating voltage of 5 kV. The nanowires were deposited on Si substrates, and prior to imaging, the samples were coated with a Pt layer to improve surface conductivity. SEM images were acquired at magnifications ranging from 1700 to 10000×.

### Optical Microscopy Measurements

Brightfield and PL imaging were conducted using an Olympus BX53 upright microscope under epi-detection mode. An X-Cite Series 120 Q Mercury lamp was used as the excitation source for brightfield imaging. A 380 nm bandpass filter (Thorlabs #FBH380-10) was added to the light path for PL imaging. A coherent continuous wave OBIS 375 nm laser excitation source was used for temperature-dependent PL. The emitted PL signal was collected using a SpectraPro HRS-300 spectrometer for spectral acquisition.

### Optically Pumped Lasing

The perovskite nanowires were optically pumped with a 400 nm laser operating at a repetition rate of 1 kHz and a pulse width of 100 fs. The pulse was generated from a frequency-doubled 800 nm Mai Tai Q-Switched Ti:Sapphire laser system. The beam was expanded through a series of lenses and directed into the microscope system. The microscope system focused the pump beam onto the nanowire samples and collected the emissions. The emissions were separated from the excitation pulse with a 520 nm dichroic mirror and directed into a grating spectrometer outfitted with both 300 and 1200 cm^–1^ blaze gratings and a liquid nitrogen cooled charge coupled device (CCD) detector. A Princeton Instruments HRS-300 spectrometer was used, providing a maximum resolution of 0.10 nm with a slit width set to 100 μm. Exposure times ranged from 100 ms to 2 s. To limit degradation of the wire, a shutter was placed in the laser excitation path to illuminate the wire only during measurements, and the sample was placed into a sealed argon chamber. Time-resolved photoluminescence above *P*_th_ was collected with a single photon detector placed at the exit slit of the spectrometer. Polarization-resolved spectra were collected by placing a polarizer at different angles in the secondary image plane of the microscope along the beam path to the spectrometer.

### Time-Resolved Photoluminescence below *P*_th_

All lifetime characterization was conducted using a custom-built scanning confocal microscope based on a commercial inverted microscope body (Nikon Ti–U), equipped with a 50 μm pinhole. Optical pumping was achieved using a 514 nm fiber-coupled diode laser (BDL-514-SMNi, Becker & Hickl) with a nominal pulse width of 100 ps and set to a repetition rate of 20 MHz. The excitation beam was reflected by a 550 nm long-pass dichroic mirror (DMLP550L, Thorlabs), and residual pump power was filtered out using a 550 nm long-pass filter (FEL0550, Thorlabs). For single-photon detection during scanning and lifetime measurements, two avalanche photodetectors (PDM, Micro-Photon Devices) were employed, each offering a 30 ps time resolution and 35% quantum efficiency at 650 nm.

### Gain Measurements

The strip-length experiment commonly described in the literature^37^ was used to measure the gain of the nanowire. A razor was attached to a precise stage and placed in the excitation beam path before the microscope. The spectrum was measured as the razor was translated across the beam. Accounting for inhomogeneities in the excitation beam, the diffraction of the beam on the razor, and different orientations, an adjusted length was implemented through measuring the overlap of the excitation beam with the wire. The gain *g* can be found according to the relation $I=I_{0}e^{gx}$ where *I* is the input intensity, *I*_0_ is constant, and *x* is the wire length that is illuminated. Accordingly, the data were plotted on a semilog plot, and a linear fit was assigned to the most linear portion of the data.

## Conclusion

The development of practical miniaturized RT lasers requires both efficient gain materials and integrated optical cavities that can be controlled and fabricated without complex or costly processing. Through our bottom-up strategy, we synthesized phase-pure quasi-2D tin halide perovskite nanowires that inherently function as FP resonators. Using a newly designed isophthalic acid-based organic spacer, namely, 5IPA3, we demonstrate that directional HOFs can simultaneously regulate crystal morphology and enhance structural rigidity. Moreover, the incorporation of the intercalants into the HOF network introduces a secondary 2D hydrogen-bonding lattice, which reorients the organic framework and facilitates the growth of well-defined optical cavities.

Despite the inherent challenges associated with Sn­(II) oxidation and typical multiphase formation at higher quantum well thickness, our method effectively suppresses phase intergrowth, enabling the synthesis of phase-pure quasi-2D tin perovskites with multiple *n*-values (*n* = 1 to 4). This advance allows a systematic investigation of lasing behavior and photophysical properties in this material class, including analysis of lasing thresholds, cavity quality factors, exciton–phonon interactions, and effective gain coefficients. Additionally, the combination of phase purity and tolerance for fabrication makes these materials an ideal platform to study optical-exciton strong coupling. While this proof of concept shows the intrinsic optical gain and structural advantages of the quasi-2D tin perovskite nanowires, further development is required to achieve continuous-wave or electrically driven lasing. These advances will necessitate improvements in carrier transport, defect passivation, and thermal management. Our findings and further explanations will enable layered tin halide perovskites as a promising platform for on-chip, solution-processable nanolasers and next-generation integrated photonic technologies.

## Supplementary Material



## Notes

Additional experimental and computational methods, extended SC-XRD characterization data, stability tests, statistical analyses, nanolasing details, temperature-dependent PL measurements, FP nanowire cavity resonance analysis, NMR spectra, and crystallographic data (PDF)
