# Supplementary material for: Hydrogen-Bonded Organic Framework Enables Phase-Pure Layered Tin Perovskite Nanowires for Room-Temperature Lasing
Source: J Am Chem Soc. 2026 Jan 2;148(1):483–93. doi: 10.1021/jacs.5c14431 (PMC12814338; doi:10.1021/jacs.5c14431)
Supplement: Supplementary file 1 [file ja5c14431_si_001.pdf]

Supporting Information

Hydrogen-bonded Organic Framework Enables Phase-pure Layered Tin Perovskite Nanowires for Room-Temperature Lasing

Jeong Hui Kim[1]†, Jeffrey Simon[2,3]†, Wenhao Shao[1,4]*, Zhichen Nian[1], Hanjun Yang[5], Peigang Chen[2,3], Brandon Triplett[2,3], Zhixu Li[1], Pengfei Wu[1], Yuheng Chen[2,3], Henna Farheen[2,3,6], Karthik Pagadala[2,3], Kyu Ri Choi[2,3,7], Colton B. Fruhling[2,3], Jens Förstner[6], Alexandra Boltasseva[2,3], Brett M. Savoie[8], Vladimir M. Shalaev[2,3]*, and Letian Dou[1,5,9]*

[1]Davidson School of Chemical Engineering, Purdue University; West Lafayette, IN 47907, USA.
[2]Elmore Family School of Electrical and Computer Engineering, Purdue University; West Lafayette, IN 47907, USA.
[3]Birck Nanotechnology Center, Purdue University; West Lafayette, IN 47907, USA.
[4]Department of Chemistry, University of Georgia; Athens, GA 30602, USA.
[5]Department of Chemistry, Purdue University; West Lafayette, IN 47907, USA.
[6]Theoretical Electrical Engineering, Institute for Photonic Quantum Systems (PhoQS), Paderborn University; Paderborn 33098, Germany.
[7]Research Institute for Nanoscale Science & Technology, Chungbuk National University; Cheongju, Chungbuk 28644, Republic of Korea
[8]Department of Chemical and Biomolecular Engineering, University of Notre Dame; Notre Dame, IN 46556, USA.
[9]Department of Chemistry, Emory University; Atlanta, GA 30322, USA.
†These authors contributed equally to this work.

*Email: swh@uga.edu (W Shao); shalaev@purdue.edu (VM Shalaev); dou10@purdue.edu (L Dou).

Methods

Synthesis of organic cations

Synthesis of 5IPA3, 2-(3,5-dicarboxyphenoxy)ethan-1-aminium iodide/bromide

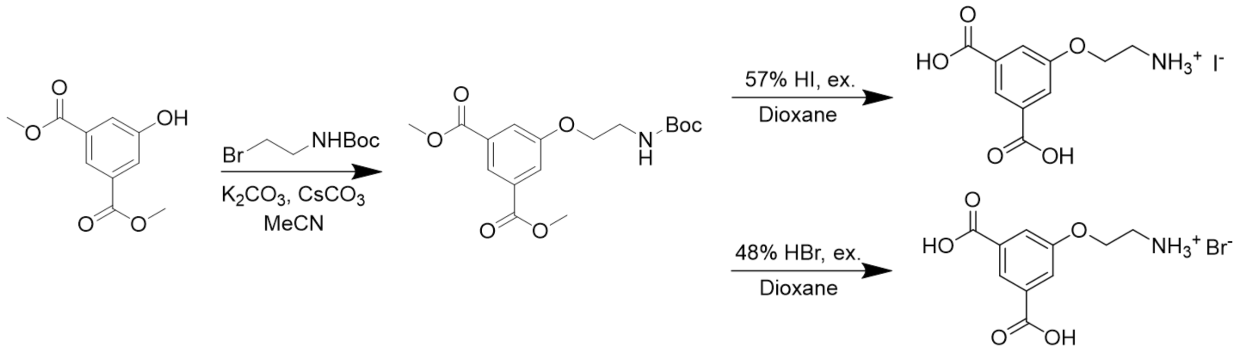

Scheme S1. Synthesis route for 5IPA3 iodide and bromide.

Step (1): Dimethyl 5-hydroxyisophthalate (from Ambeed, 10 mmol, 1.0 equiv.), potassium carbonate (K_2CO_3, from Millipore Sigma, 20 mmol, 2.0 equiv.), cesium carbonate (Cs_2CO_3, from Millipore Sigma, 5 mmol, 0.5 equiv.), and *tert*-butyl (2-bromoethyl)carbamate (from Ambeed, 20 mmol, 2.0 equiv.) were placed in a round-bottom flask (RBF) with a magnetic stir bar. The flask was sealed with a rubber septum, and purged with argon briefly. Anhydrous acetonitrile (MeCN, Millipore Sigma, 50 mL) was then added via syringe. After extensive purging with Ar, the reaction mixture was stirred and refluxed at 90 °C for 12 hours. Upon completion, the reaction mixture was cooled to room temperature.

Water was added to the mixture, and the crude product was extracted with chloroform. The organic layers were combined, dried over magnesium sulfate, and filtered. The residue was subjected to purification by silica gel column chromatography using a hexane/ethyl acetate gradient. Notably, product and starting material often exhibit similar R_f values, making them difficult to separate by chromatography. The residual starting material present in the product can be effectively removed in a subsequent purification step following conversion of the product to its corresponding ammonium salt.

Step (2): To an RBF equipped with a magnetic stir bar, the Boc-protected amine obtained from the previous step (1 mmol, 1.0 equiv.) was added, followed by anhydrous 1,4-dioxane (from Millipore

Sigma, 0.2 M). After the compound fully dissolved, 57% aqueous hydroiodic acid (from Millipore Sigma, 10 mmol, 10 equiv.) was added for iodide salt formation, or 48% aqueous hydrobromic acid (from Millipore Sigma, 10 mmol, 10 equiv.) for bromide salt formation. The RBF and condenser were thoroughly purged with argon, and the mixture was stirred and refluxed at 90 °C for 12 hours. Upon completion, the reaction mixture was cooled to room temperature.

Solvents, including residual water from the aqueous HBr or HI, were completely removed by rotary evaporation under reduced pressure. The resulting crude residue was then reprecipitated at least three times by dissolving in acetone, followed by the slow addition of diethyl ether to induce precipitation.

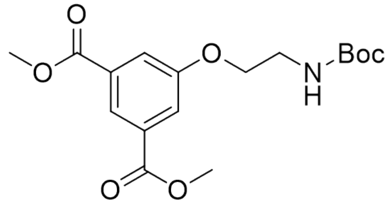

5IPA3-Boc

(5IPA3-Boc), dimethyl 5-(2-((tert-butoxycarbonyl)amino)ethoxy)isophthalate

- Materials: dimethyl 5-hydroxyisophthalate (10 mmol, 1 eq.), K_2CO_3 (20 mmol, 2 eq.), Cs_2CO_3 (5 mmol, 0.5 eq.), *tert*-butyl (2-bromoethyl)carbamate (20 mmol, 2 eq.), anhydrous MeCN (50 mL).
- Final product: transparent liquid with 19 mol% dimethyl 5-hydroxyisophthalate. Yield: 68%.
- ^{1}H NMR (400 MHz, $CDCl_3$) δ 8.27 (s, Ph-H, 1H), 7.74 (s, Ph-H, 2H), 4.99 (s, -NH, 1H), 4.11 (m, O-CH_2-, 2H), 3.83 (s, -O-CH_3, 6H), 3.25 (m, CH_2-NH, 2H), 1.46 (s, -Boc, 9H).

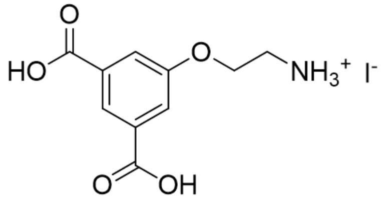

5IPA3-I

(5IPA3-I), 2-(3-carboxy-5-(methoxycarbonyl)phenoxy)ethan-1-aminium iodide

- Materials: 5IPA3-Boc (1 mmol, 1 eq.), anhydrous dioxane (0.2 M), HI (10 mmol, 10 eq.).

- Product reprecipitated with acetone/ether.
- Product: pale yellow powder. Yield: 85%
- ^{1}H NMR (400 MHz, DMSO-d6) δ 13.3 (s, -COOH, 2H), 8.11 (s, Ph-H, 1H), 7.97 (s, -NH_3, 2H), 7.71 (s, Ph-H, 2H), 4.28 (m, O-CH_2-, 2H), 3.25 (m, CH_2-NH_3, 2H).
- ^{13}C NMR (126 MHz, DMSO-d6) δ 166.6, 158.4, 133.3, 123.2, 119.9, 65.4, 38.6.

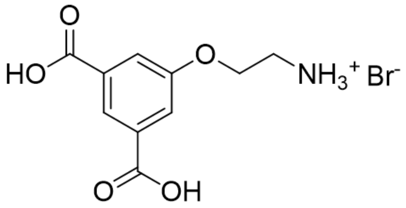

5IPA3-Br

<u>**(5IPA3-Br),** 2-(3-carboxy-5-(methoxycarbonyl)phenoxy)ethan-1-aminium bromide</u>

- Materials: 5IPA3-Boc (1 mmol, 1 eq.), anhydrous dioxane (0.2 M), HBr (10 mmol, 10 eq.).
- Product reprecipitated with acetone/ether.
- Product: white powder. Yield: 90%
- ^{1}H NMR (400 MHz, DMSO-d6) δ 13.3 (s, -COOH, 2H), 8.12 (s, Ph-H, 1H), 7.98 (s, -NH_3, 2H), 7.7 (s, Ph-H, 2H), 4.28 (m, O-CH_2-, 2H), 3.25 (m, CH_2-NH_3, 2H).
- ^{13}C NMR (126 MHz, DMSO-d6) δ 166.6, 158.4, 133.3, 123.2, 119.8, 65.2, 38.6.

Table S1. Precursor solutions for perovskite crystal growth

Composition	Cation (mg)	SnI_2 (mg)	MAI (mg)	HI/H_3PO_2 (µL)	Acetic acid (µL)5	Yield (%)
$(5IPA3)_2SnI_4$ (n = 1)	2.0	20.0	-	50/25	37.6	64.9
$(5IPA3)_2MASn_2I_7$ (n = 2)	2.0	5.0	1.5	50/25	37.6	66.5
$(5IPA3)_2MA_2Sn_3I_{10}$ (n = 3)	2.0	10.0	1.5	50/25	37.6	55.2
$(5IPA3)_2MA_3Sn_4I_{13}$ (n = 4)	1.0	10.0	7.5	66/33	50	57.2

Composition	Cation (mg)	$PbBr_2$ (mg)	MAI (mg)	HBr (µL)	Yield (%)
$(TPA3)_2PbBr_4$	0.5	20.0	-	300	
$(5IPA3)_2PbBr_4$	1.2	20.1	-	350	77.0

Computational methods

Binding energy (E_B) calculation

The in-plane binding energy was calculated as:

$$E_B = \frac{E_{bulk} - E_{slab}}{A}$$

where E_{bulk} was the total energy for the bulk crystal, and the E_{slab} is the energy of surface slab, The energy value was normalized with respect to the surface area.

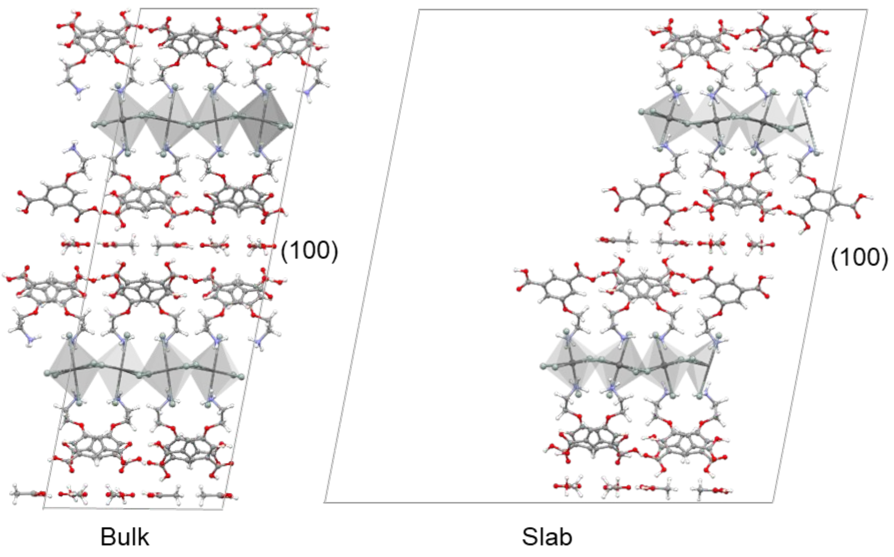

Figure S1. Schematics of full unit cell and its corresponding in-plane surface slab. Binding energy is calculated based on the equation shown above.

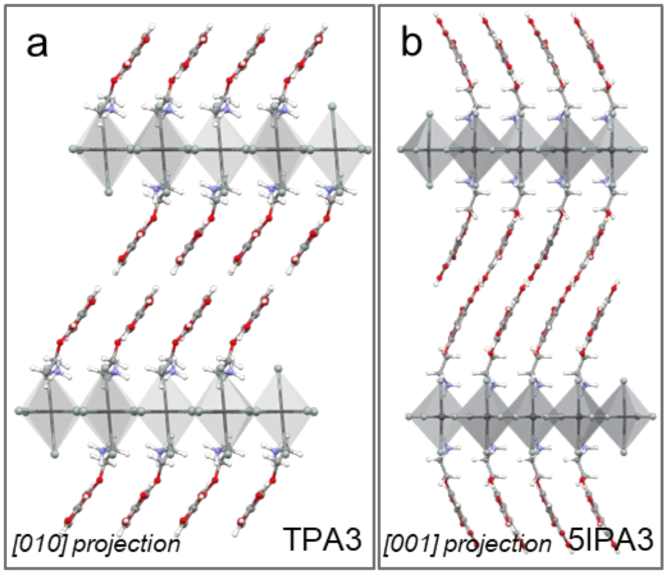

Figure S2. Structural comparison of $(TPA3)_2PbBr_4$ and $(5IPA3)_2PbBr_4$. (a) Crystal structure of $(TPA3)_2PbBr_4$ along [010] and **(b)** $(TPA3)_2PbBr_4$ along [001].

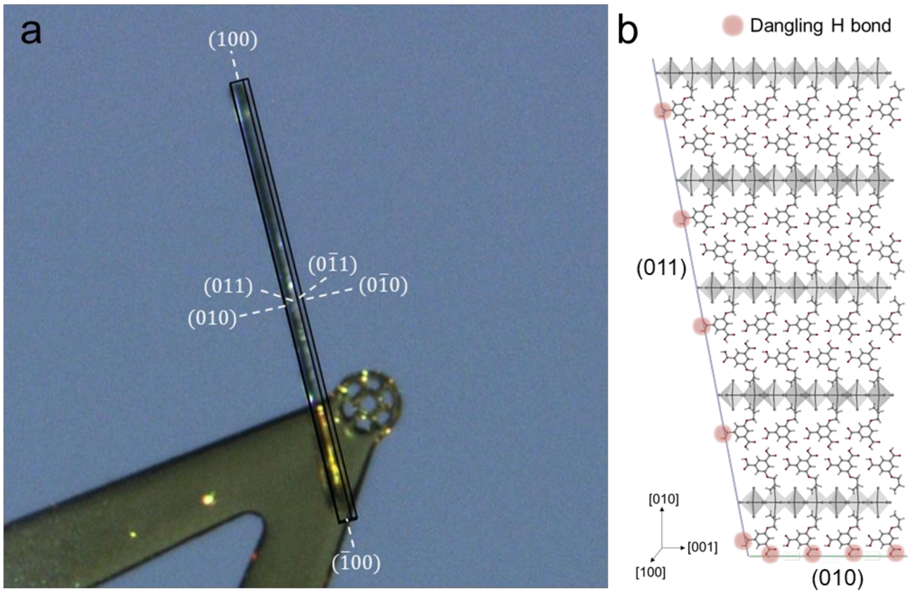

Figure S3. Face indexing of $(5IPA3)_2PbBr_4$ nanowire. (a) A bulk $(5IPA3)_2PbBr_4$ crystal with side faces indexed by SC-XRD. **(b)** Cross-section view along [100] from $(5IPA3)_2PbBr_4$ crystal structure. Red clouds represent the surface dangling COOH.

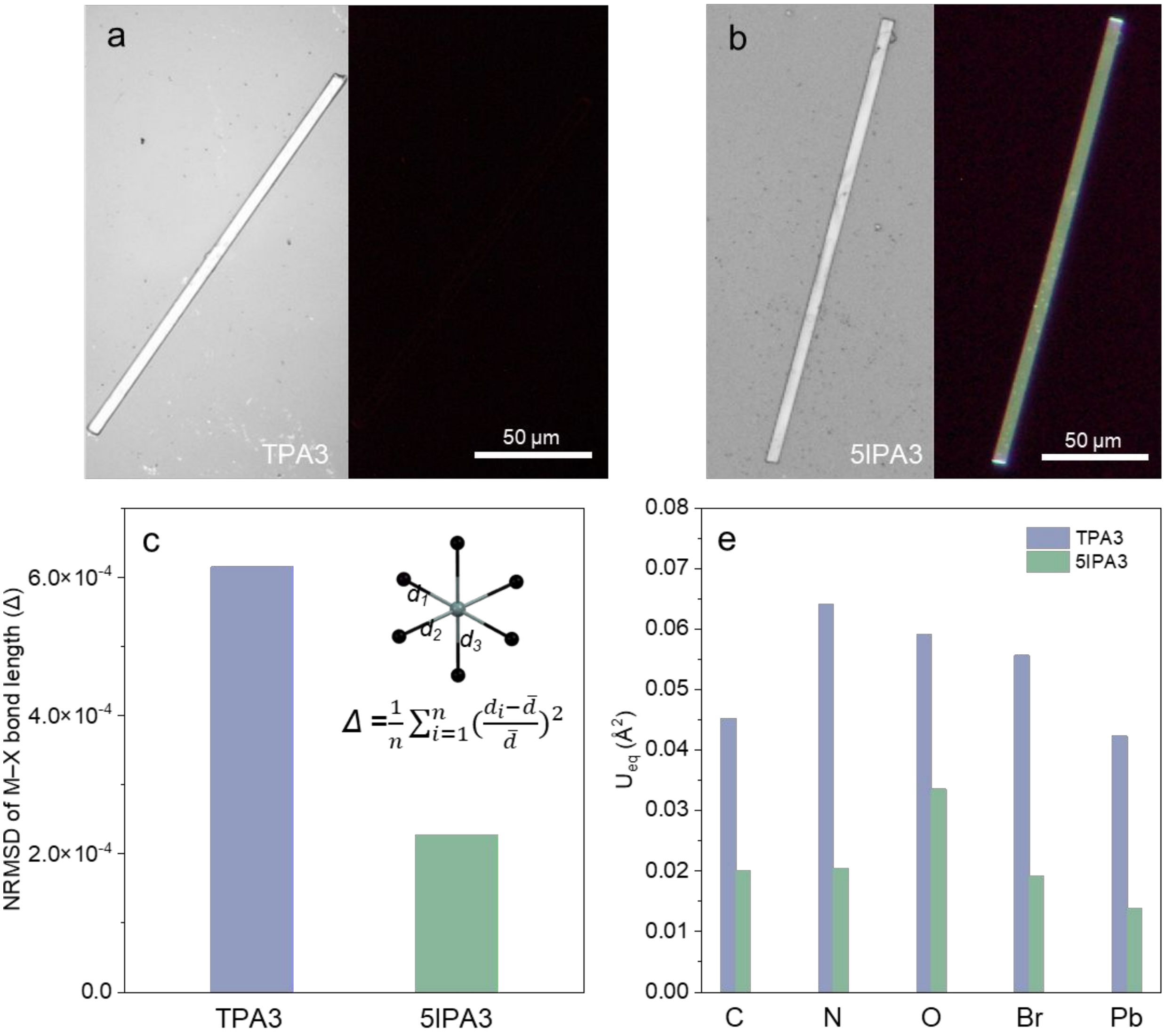

Figure S4. Optical property comparison depending on octahedral distortion. Bright field image and PL image of **(a)** $(TPA3)_2PbBr_4$ and **(b)** $(5IPA3)_2PbBr_4$ nanowires. **(c)** Normalized root-mean-square distance (NRMSD) of the metal (M) - halide (X) bond (Δ) analyzed from the $(TPA3)_2PbBr_4$ and $(5IPA3)_2PbBr_4$ crystal structures. **(d)** The equivalent isotropic displacement parameters (Ueq) extracted from refined crystal structure CIF files.

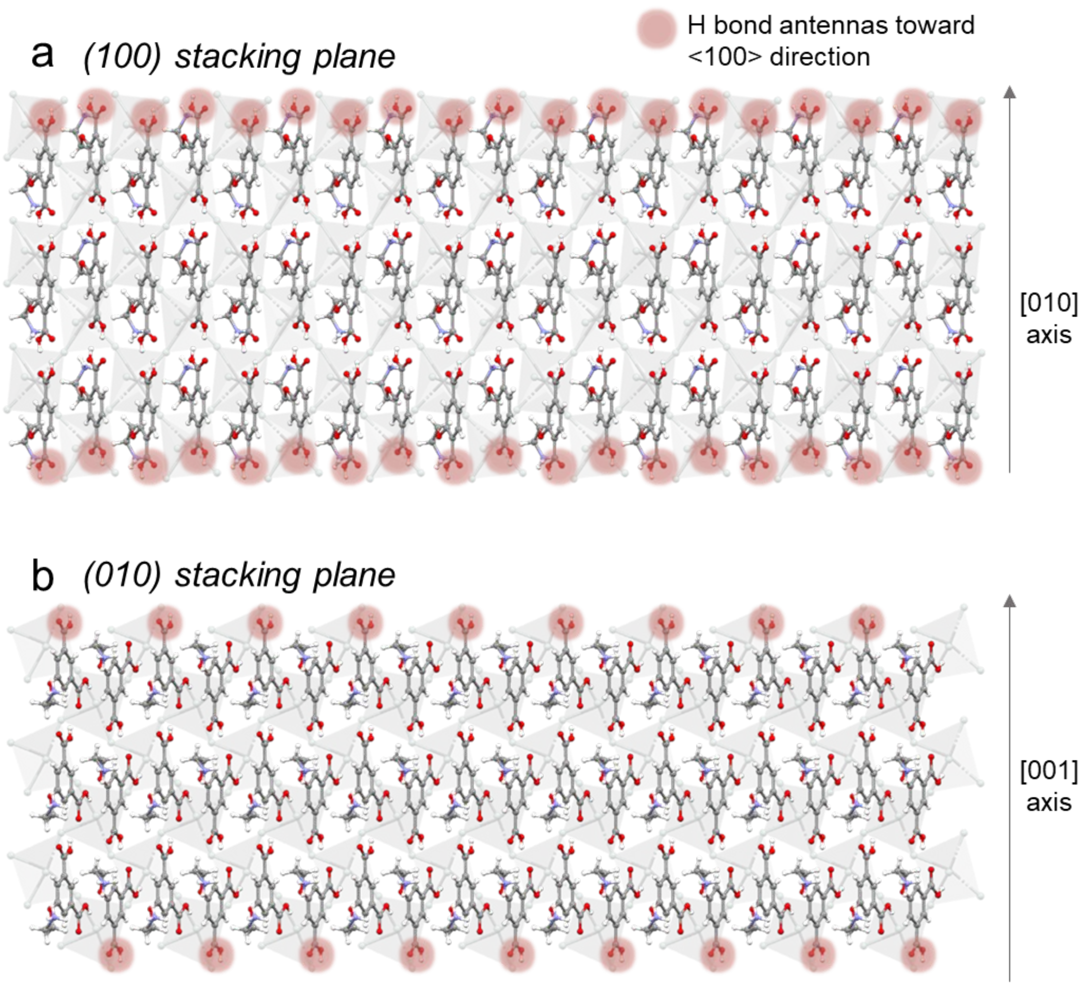

Figure S5. Cross-section view of the crystal structures. (a) $(TPA3)_2PbBr_4$ structure at (100) plane shows COOH along [010] axis. **(b)** $(5IPA3)_2PbBr_4$ structure at (010) plane reveals COOH along [001] axis.

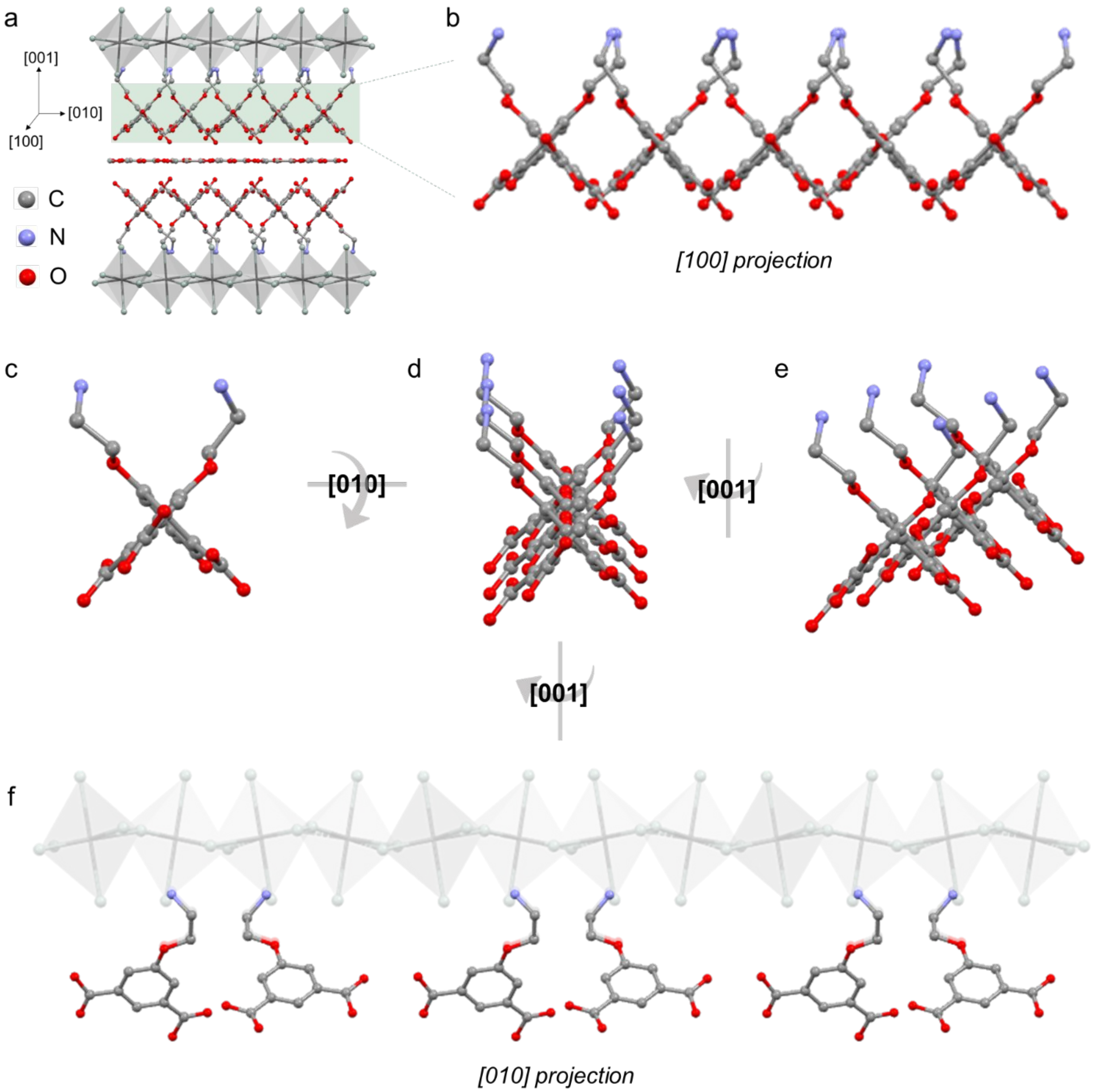

Figure S6. Detailed view of organic spacer layer in the intercalated $(5IPA3)_2SnI_4$ crystal. (a) Crystal structure along [100] direction. **(b)** [100] projection of organic spacer layer. **(c)** Part of organic spacer layer from (b). **(d)** Rotation of 3° about the [010] axis relative to (c). **(e)** Rotation of 6° about the [001] axis relative to (d). **(f)** Rotation of 90° about the [001] axis relative to (d). Hydrogen atoms were omitted for clarity.

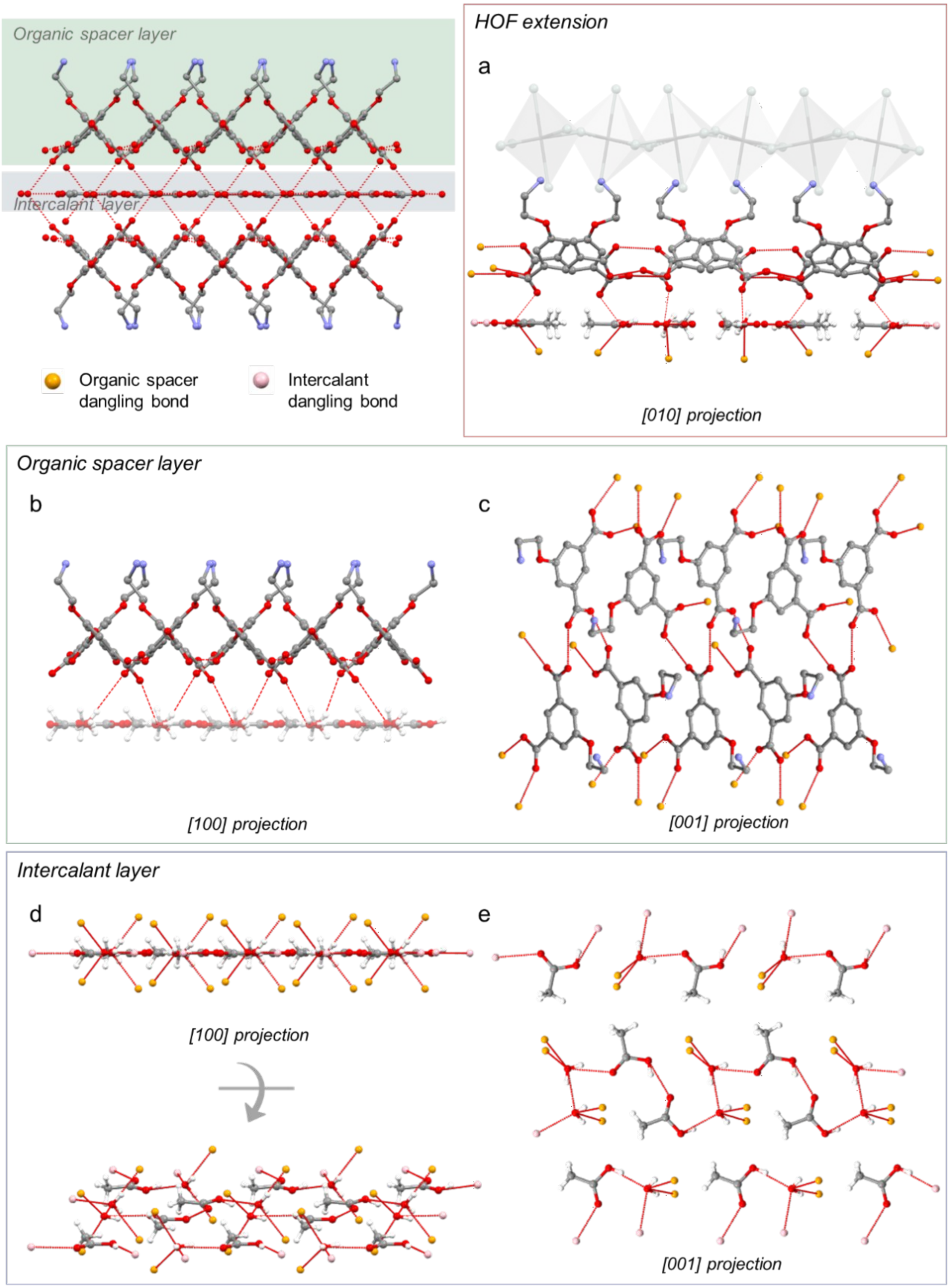

Figure S7. Detailed view of hydrogen bonds in the intercalated $(5IPA3)_2SnI_4$ crystal. (a) Crystal structure along [010] direction to explain HOF extension. Hydrogen bonds in organic spacer layer viewed along **(b)** [100] and **(c)** [001]. Hydrogen bonds in intercalant layer viewed along **(d)** [100] and **(e)** [001].

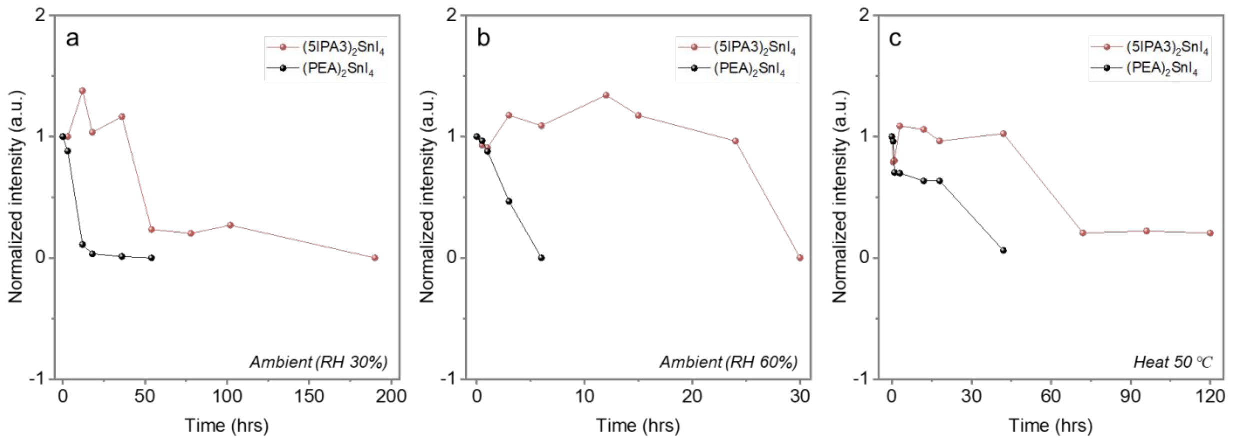

Figure S8. Stability comparison between $(5IPA3)_2SnI_4$ nanowires and $(PEA)_2SnI_4$ flakes based on integrated PL. (a) in air at RH 30%, **(b)** in air at RH 60%, and **(c)** in N_2 at 50 ℃.

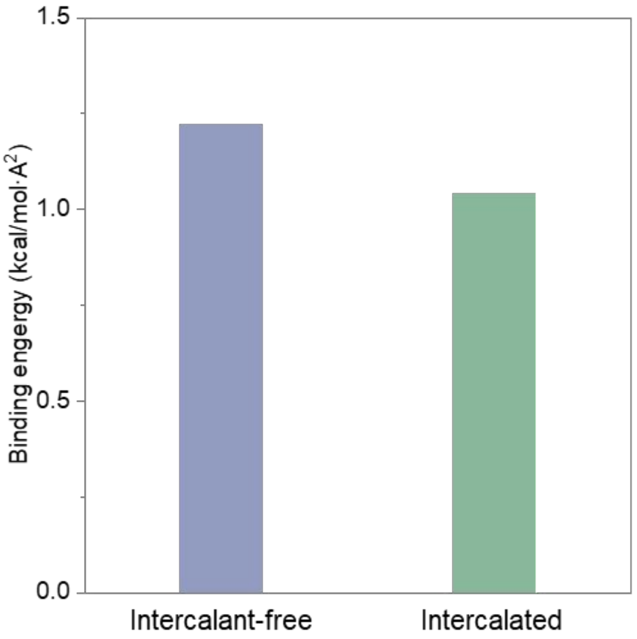

Figure S9. Binding energies between "Intercalant-free" and "Intercalated" $(5IPA3)_2SnI_4$ structures. Computational details are described in the computational method section.

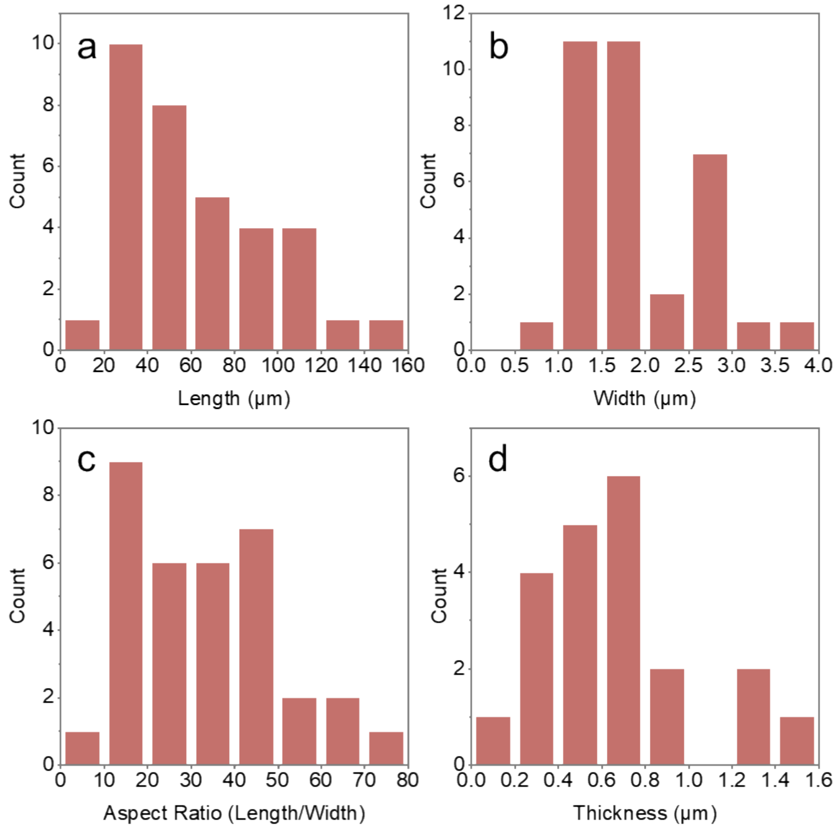

Figure S10. Population statistics of $(5IPA3)_2SnI_4$ nanowires. (a) Length, (b) Width, (c) Aspect ratio (length/width), and (d) Thickness distribution.

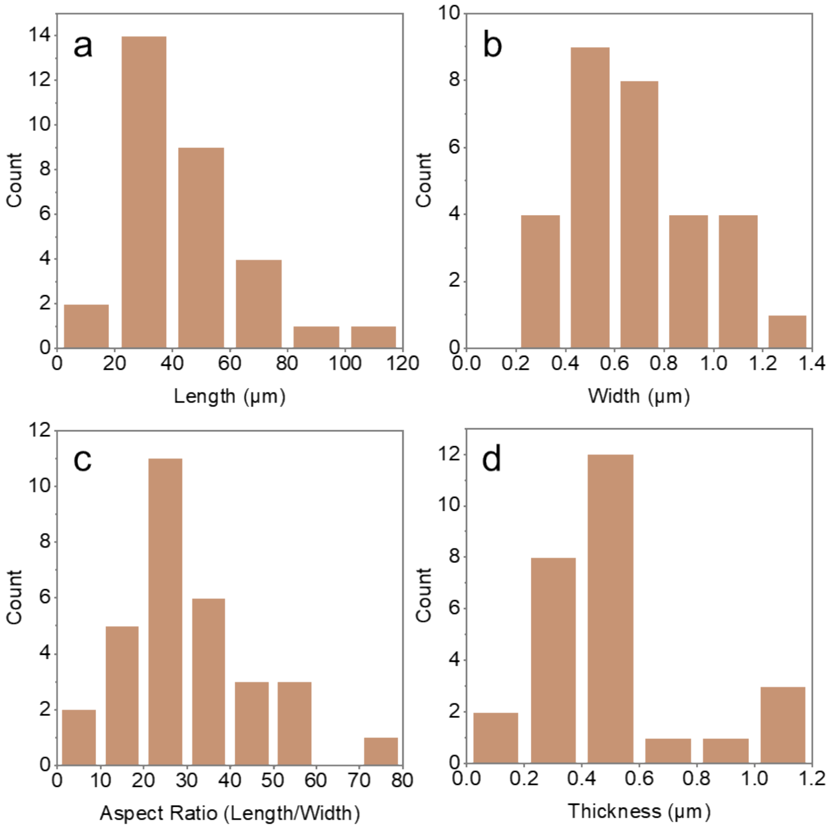

Figure S11. Population statistics of $(5IPA3)_2(MA)Sn_2I_7$ nanowires. (a) Length, (b) Width, (c) Aspect ratio (length/width), and (d) Thickness distribution.

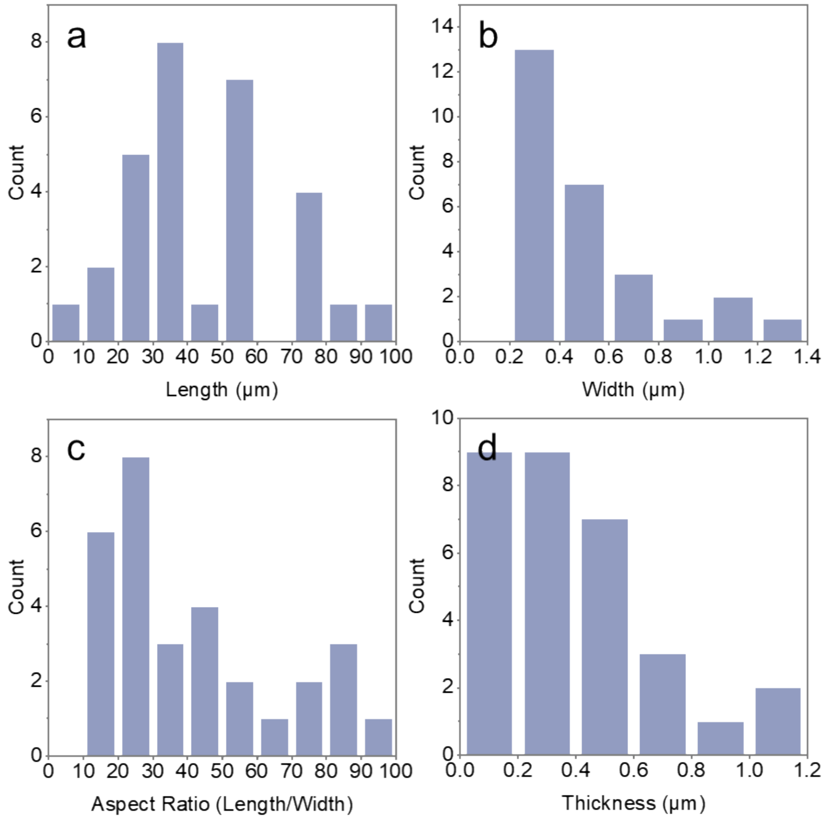

Figure S12. Population statistics of $(5IPA3)_2(MA)_2Sn_3I_{10}$ nanowires. (a) Length, (b) Width, (c) Aspect ratio (length/width), and (d) Thickness distribution.

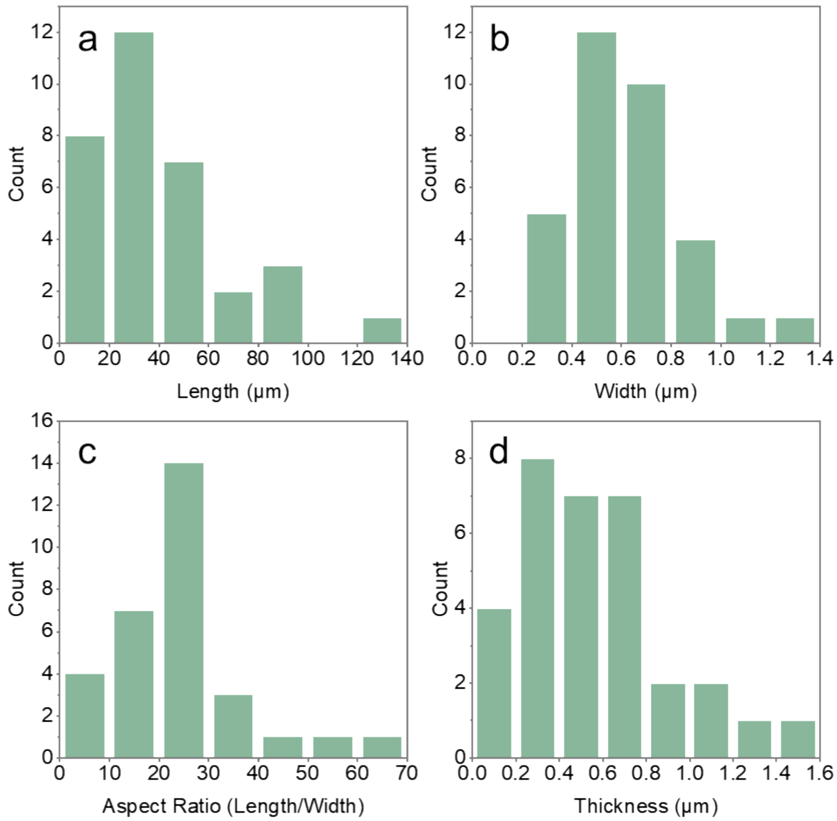

Figure S13. Population statistics of $(5IPA3)_2(MA)_3Sn_4I_{13}$ nanowires. (a) Length, (b) Width, (c) Aspect ratio (length/width), and (d) Thickness distribution.

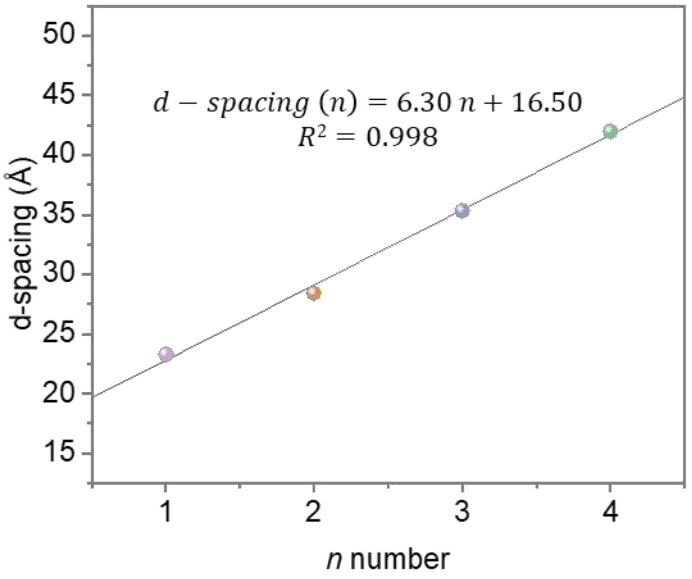

Figure S14. Linear regression of *d*-spacing versus *n* number from perovskite nanowire PXRD. *d*-spacing values were obtained from spatially random nanowire domains, and fitted using the linear equation, where 6.30 Å is the spacing increment.

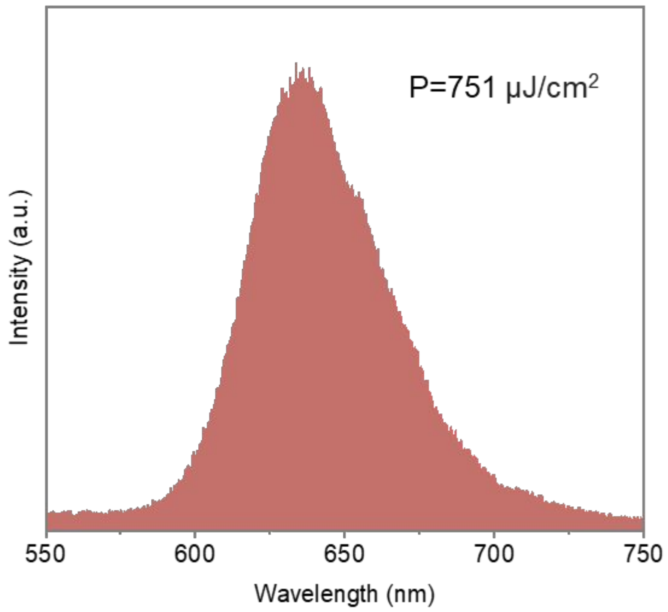

Figure S15. Emission spectrum of $(5IPA3)_2SnI_4$ (n = 1) under a high pump fluence, collected using a 300 gr/mm grating. Photoluminescence spectrum collected at a pump fluence of 751 µJ/cm^2. Lasing was not observed.

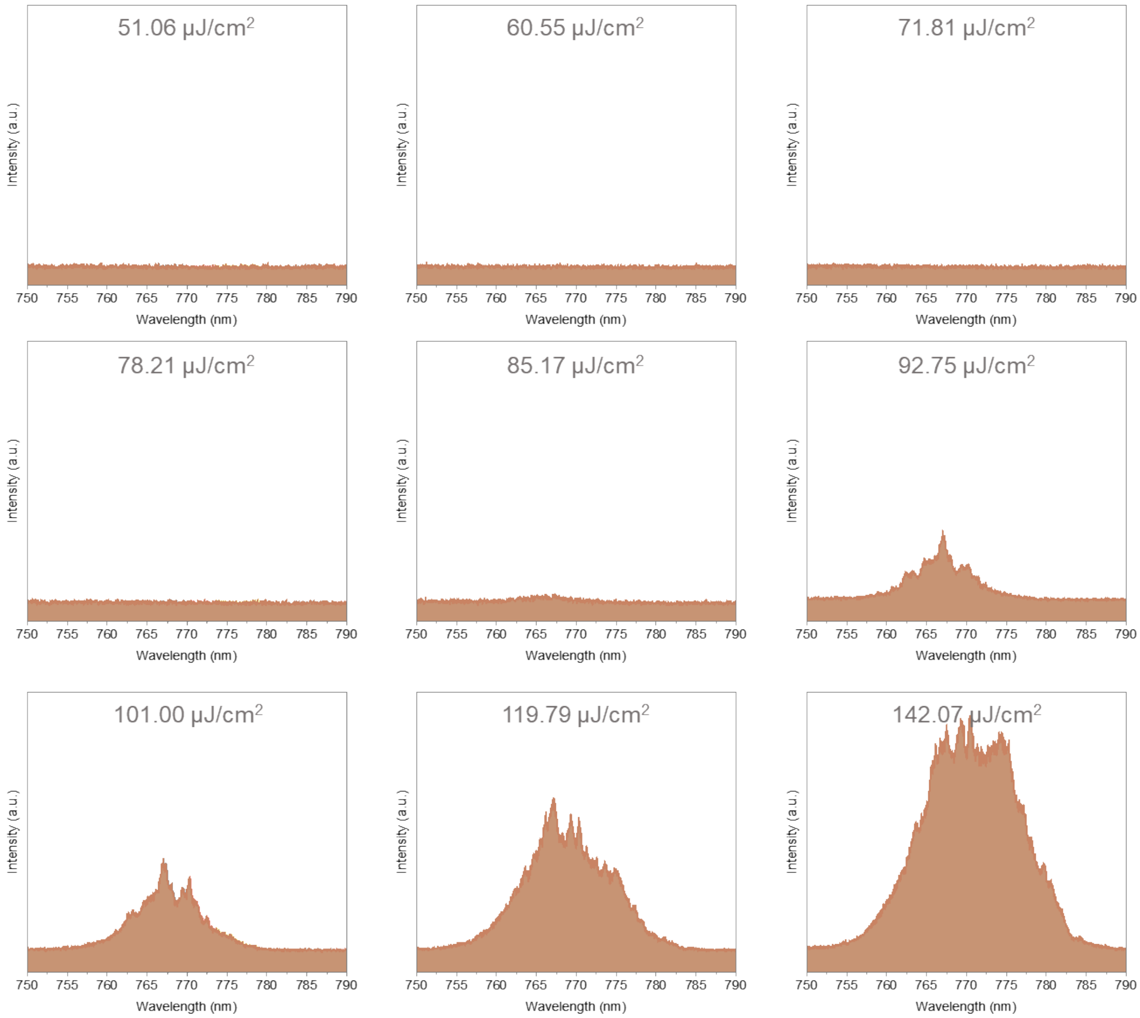

Figure S16. Progression of nanolasing at room-temperature (n = 2). PL spectra (1200 gr/mm grating) with increasing pump fluence including across the lasing threshold of the $(5IPA3)_2(MA)Sn_2I_7$ (n = 2) nanowire examined in Figure 3a (P_{th}: 92.8 µJ/cm^2).

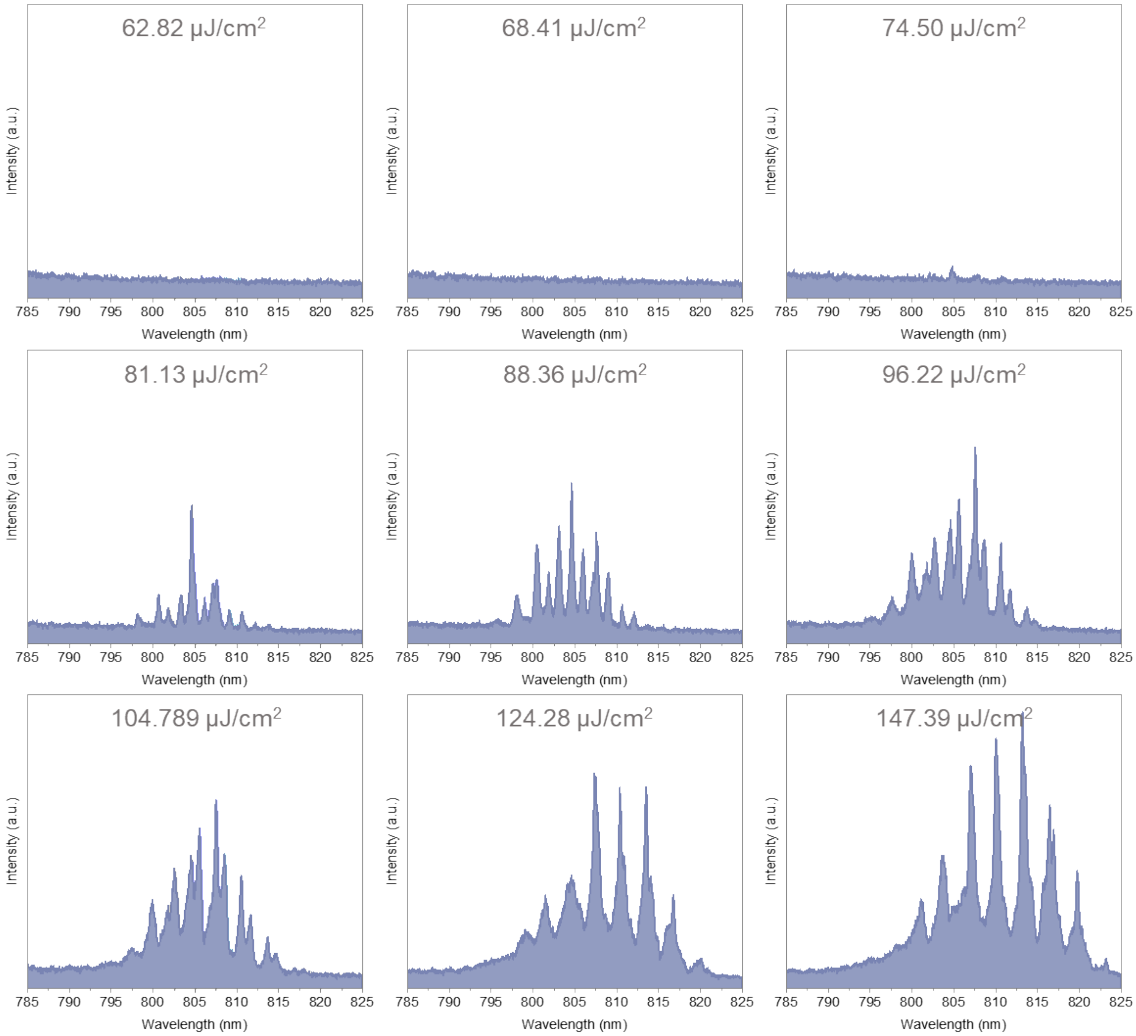

Figure S17. Progression of nanolasing at room-temperature (n = 3). PL spectra (1200 gr/mm grating) with increasing pump fluence including across the lasing threshold of the $(5IPA3)_2(MA)_2Sn_3I_{10}$ (n = 3) nanowire examined in Figure 3a (P_{th}: 75.8 μJ/cm^2).

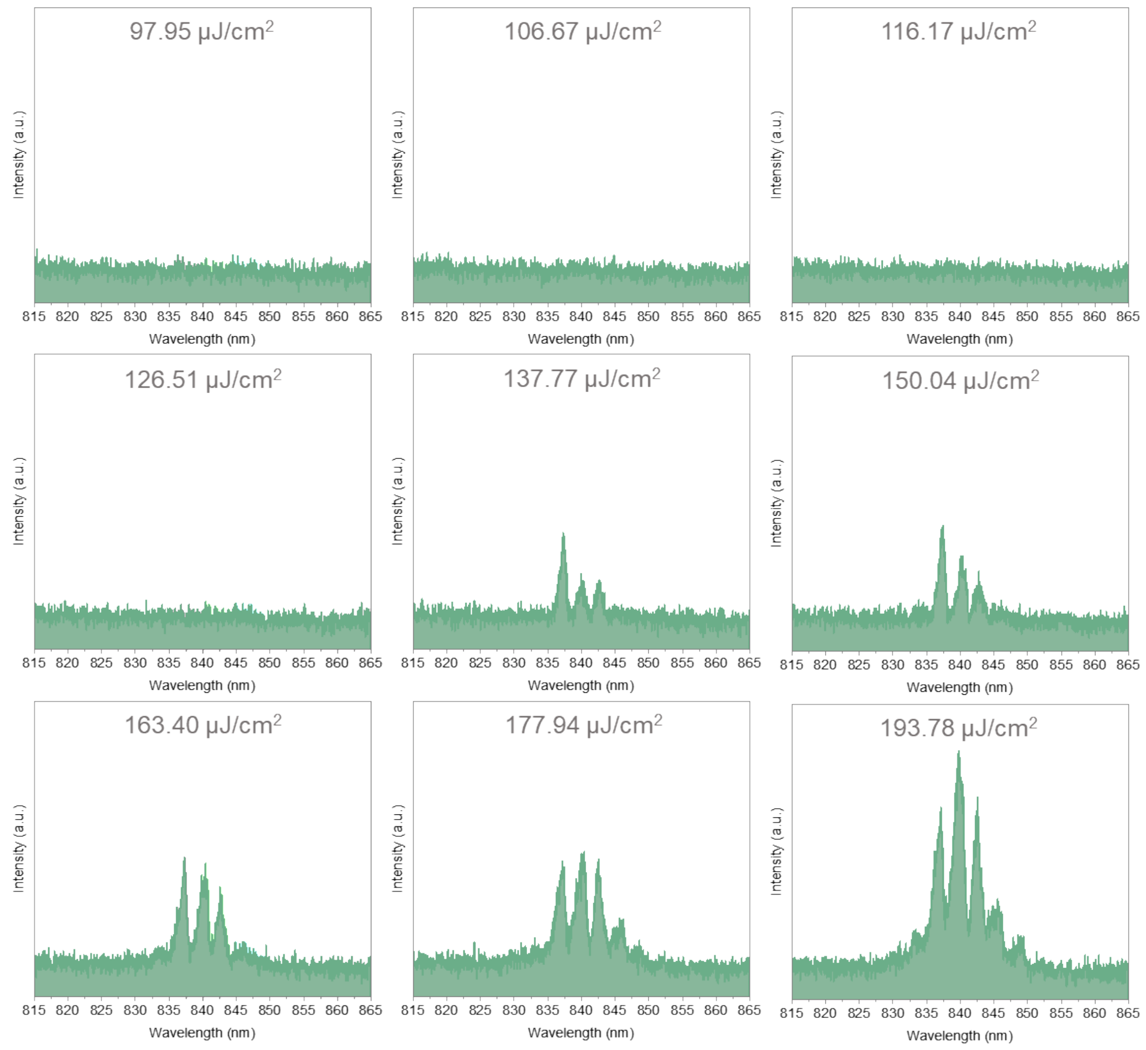

Figure S18. Progression of nanolasing at room-temperature (n = 4). PL spectra (1200 gr/mm grating) with increasing pump fluence including across the lasing threshold of the $(5IPA3)_2(MA)_3Sn_4I_{13}$ (n = 4) nanowire examined in Figure 3a (P_{th}: 131.0 μJ/cm^2).

Table S2. PL lifetimes below and above threshold (P_{th}) for n = 2, 3, and 4 nanowires. Extracted from a bi-exponential fitting of TRPL shown in Figure 3b.

$$I(t) = A_1 e^{-\frac{t}{\tau_1}} + A_2 e^{-\frac{t}{\tau_2}}$$

$$\tau_{avg} = \frac{A_1\tau_1^2 + A_2\tau_2^2}{A_1\tau_1 + A_2\tau_2}$$

	A_1	τ_1 (ns)	A_2	τ_2 (ns)	τ_{avg} (ns)
n = 2 below P_{th}	0.877	0.57	0.123	4.48	2.62
n = 2 above P_{th}	1.001	0.32	0.006	4.40	0.63
n = 3 below P_{th}	0.945	0.44	0.046	4.58	1.83
n = 3 above P_{th}	0.761	0.28	0.383	0.28	0.28
n = 4 below P_{th}	0.844	0.79	0.156	4.73	2.86
n = 4 above P_{th}	1.262	0.22	0.040	0.29	0.22

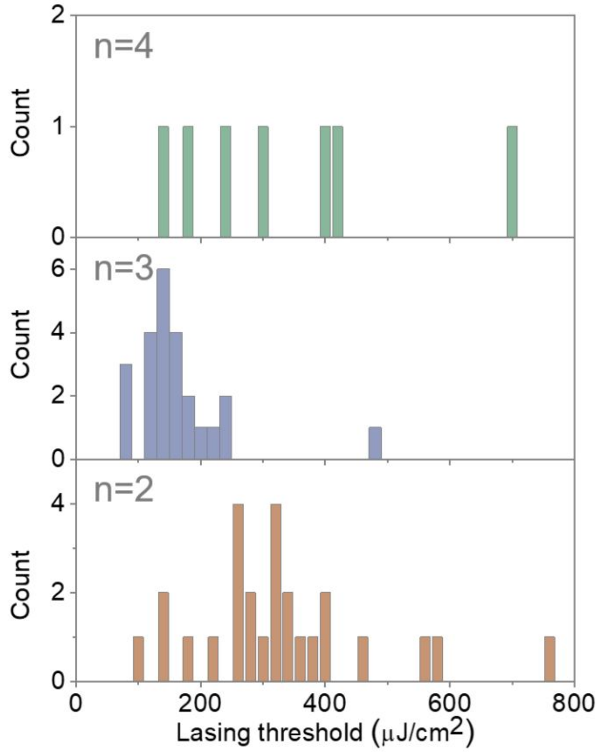

	Sample size	Best threshold (P_{th}) (μJ/cm²)	Mean threshold ($\bar{P}_{th}$) (μJ/cm²)
**n=2**	24	92.8	329.1
**n=3**	27	75.8	163.3
**n=4**	7	131.0	340.3

Figure S19. Lasing threshold statistics for n = 2, 3, and 4 nanowires. Statistical distribution of lasing thresholds (P_{th}) extracted from power-dependent PL of multiple nanowires for each *n* number.

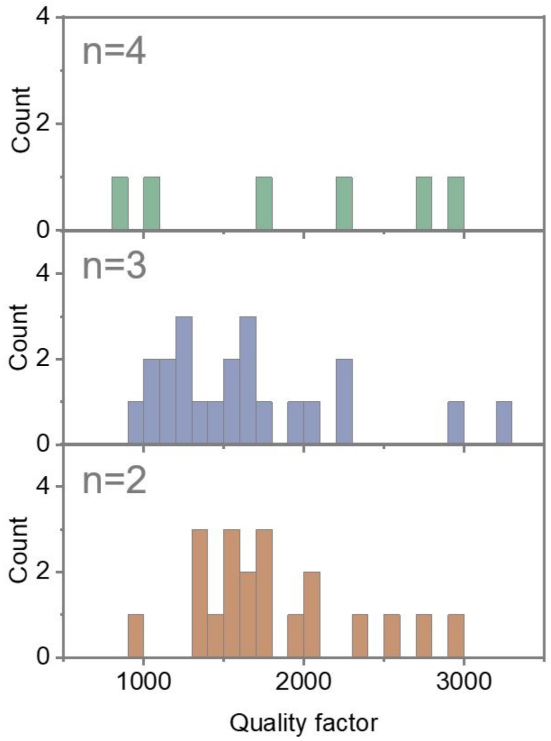

	Sample size	Max quality factor (Q)	Mean quality factor ($\bar{Q}$)
**n=2**	20	2971	1818
**n=3**	22	3276	1663
**n=4**	6	2918	1933

Figure S20. Lasing quality factor statistics for n = 2, 3, and 4 nanowires. Statistical distribution of quality factor (Q) extracted from above-threshold PL spectra of multiple nanowires for each *n* number. Q was calculated from the position and width of narrowest lasing peak, found through fitting with a Lorentzian function.

Table S3. Comparative summary of reported phase-pure 2D and quasi-2D perovskite single crystal lasers.

Materials	Lasing wavelength	Temperature	Threshold	Quality factor	Reference
$(BA)_2(MA)_2Pb_3I_{10}$	621 nm	78 K	2.6 μJ/cm^2	-	Ref 1[1]
$(PEA)_2(MA)Pb_2I_7$	598 nm	78 K	60 μJ/cm^2 (1.2 GW/ cm^2)	-	Ref 2[2]
$(2T)_2(MA)_2Pb_3I_{10}$	~ 625 nm	150 K	16 μJ/cm^2	-	Ref 3[3]
$(3T)_2(MA)_2Pb_3I_{10}$	~ 625 nm	150 K	13.2 μJ/cm^2	-	Ref 3
$(3T)_2(MA)Sn_2I_7$	729 nm	83 K	11.2 μJ/cm^2	1869	Ref 4[4]
$(3T)_2(MA)Sn_2I_7$	729 nm	RT	374 μJ/cm^2	-	Ref 4
$(BrCA3)_2SnI_4$	~ 630 nm	88 K	16.96 μJ/cm^2	-	Ref 5[5]
$(PMA)_2(MA)Pb_2I_7$	587 nm	80 K	9.2 μJ/cm^2	1500	Ref 6[6]
$(5IPA3)_2(MA)Sn_2I_7$	763 nm	RT	92.8 μJ/cm^2	2971	This work
$(5IPA3)_2(MA)_2Sn_3I_{10}$	801 nm	RT	75.8 μJ/cm^2	3276	This work
$(5IPA3)_2(MA)_3Sn_4I_{13}$	865 nm	RT	131.0 μJ/cm^2	2918	This work

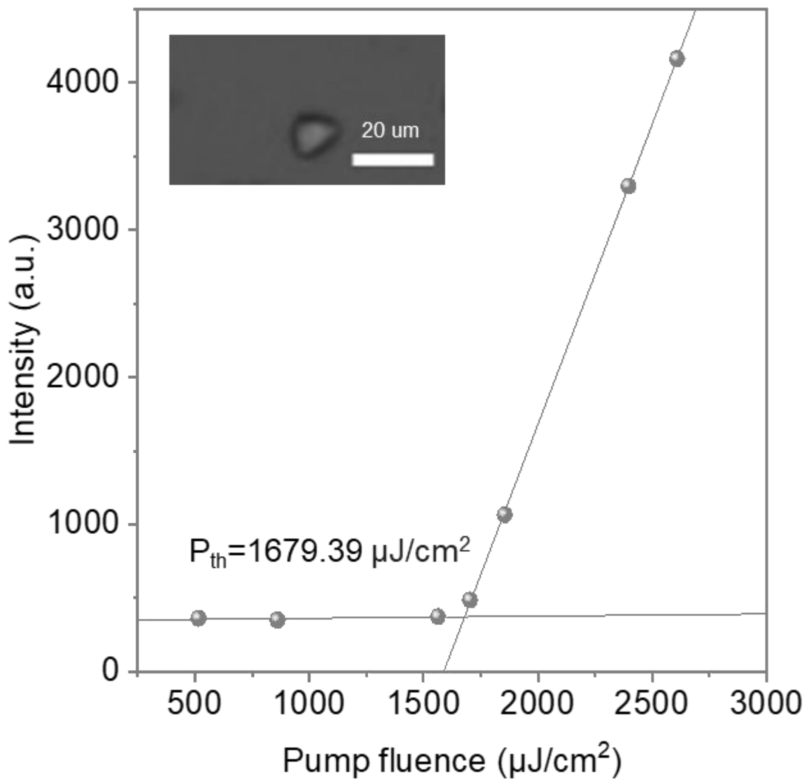

Figure S21. Input-output power characteristics showing the non Fabry–Pérot mode lasing threshold of a $(5IPA3)_2(MA)_2Sn_3I_{10}$ (n = 3) particle. Emission intensity as a function of pump fluence reveals a distinct lasing threshold, despite the absence of a conventional Fabry-Pérot cavity.

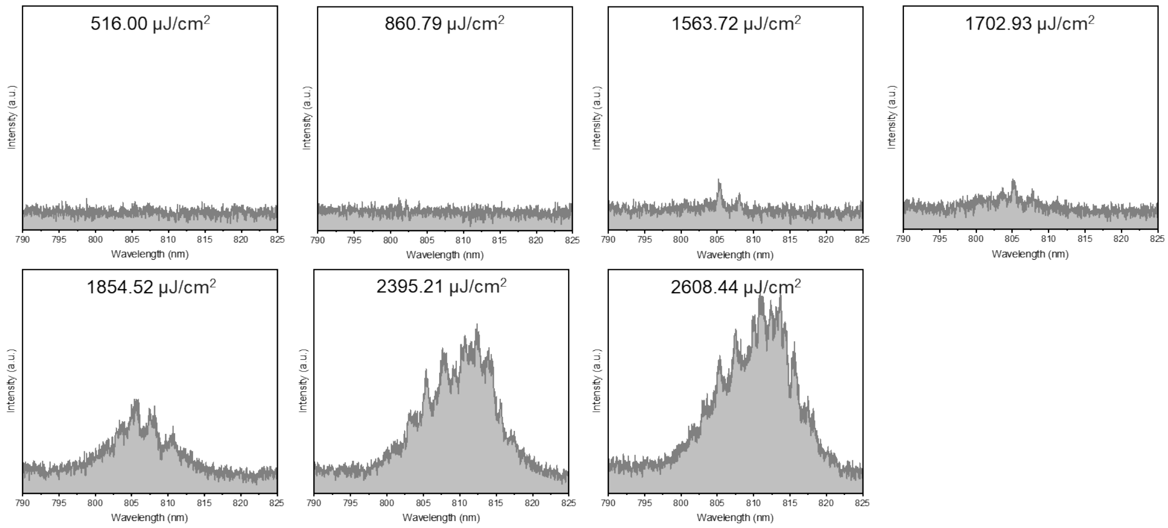

Figure S22. Progression of nanolasing at room-temperature (n = 3). PL spectra (1200 gr/mm grating) with increasing pump fluence across the threshold of the non Fabry–Pérot mode $(5IPA3)_2(MA)_2Sn_3I_{10}$ (n = 3) examined in Figure S14 (P_{th}: 1679.39 μJ/cm^2).

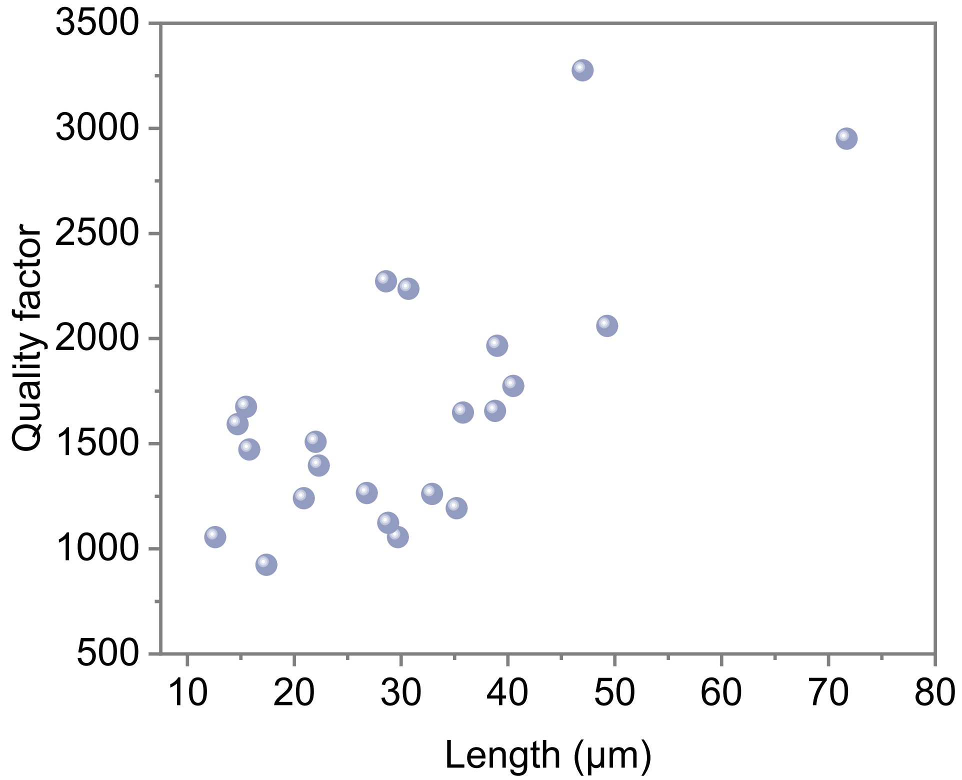

Figure S23. Quality factor-length dependence in n = 3 nanowires. A linear correlation is observed between the cavity length and the quality factor, consistent with Fabry-Pérot optical resonances. While not plotted, we note the trend is not as clear for the n = 2 and n = 4 nanowires.

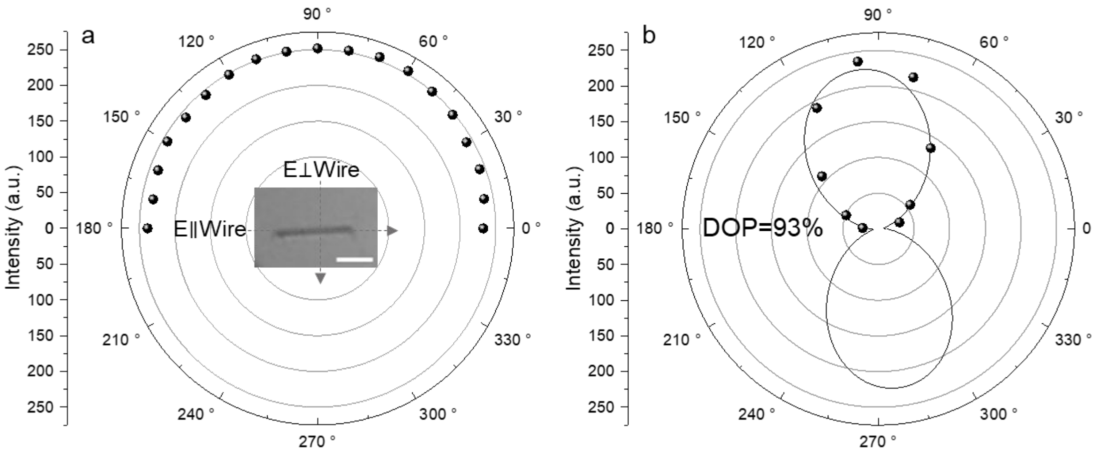

Figure S24. Linearly polarized PL from a $(5IPA3)_2(MA)_2Sn_3I_{10}$ (n = 3) nanowire below and above the lasing threshold. (a) Spontaneous emission without measurable polarization. **(b)** Linearly polarized lasing with a high DOP of 93%. (Scale bar: 10 μm)

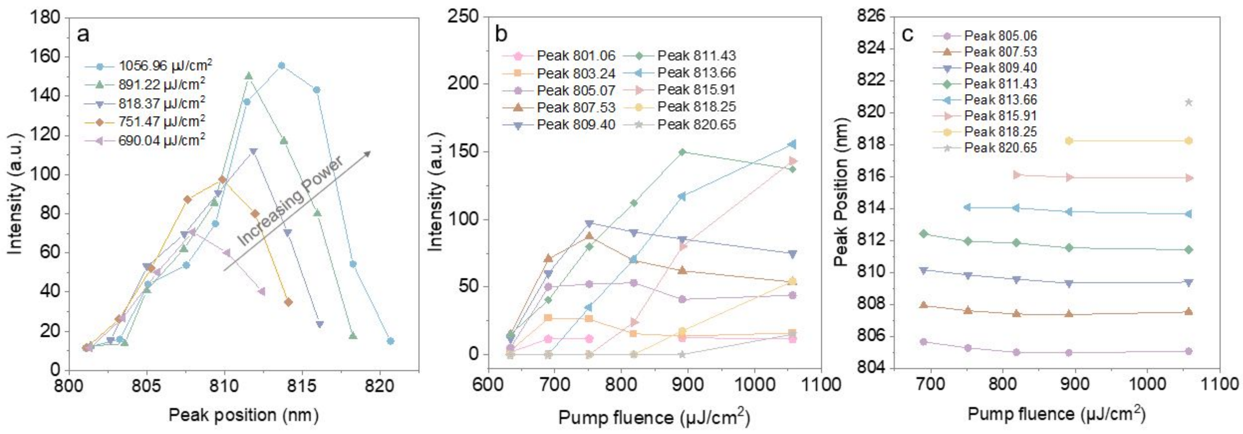

Figure S25. Power-dependent spectral response of lasing modes in a $(5IPA3)_2(MA)_2Sn_3I_{10}$ (n = 3) nanowire. The amplitude and center wavelength of each lasing mode was determined through peak fitting with Lorentzian lineshapes. **(a)** The peak amplitudes plotted with respect to their position to display the lasing spectrum envelope at various pump powers. **(b)** Output power versus input pump power for each lasing mode. **(c)** Dependence of the lasing mode wavelengths on pump fluence.

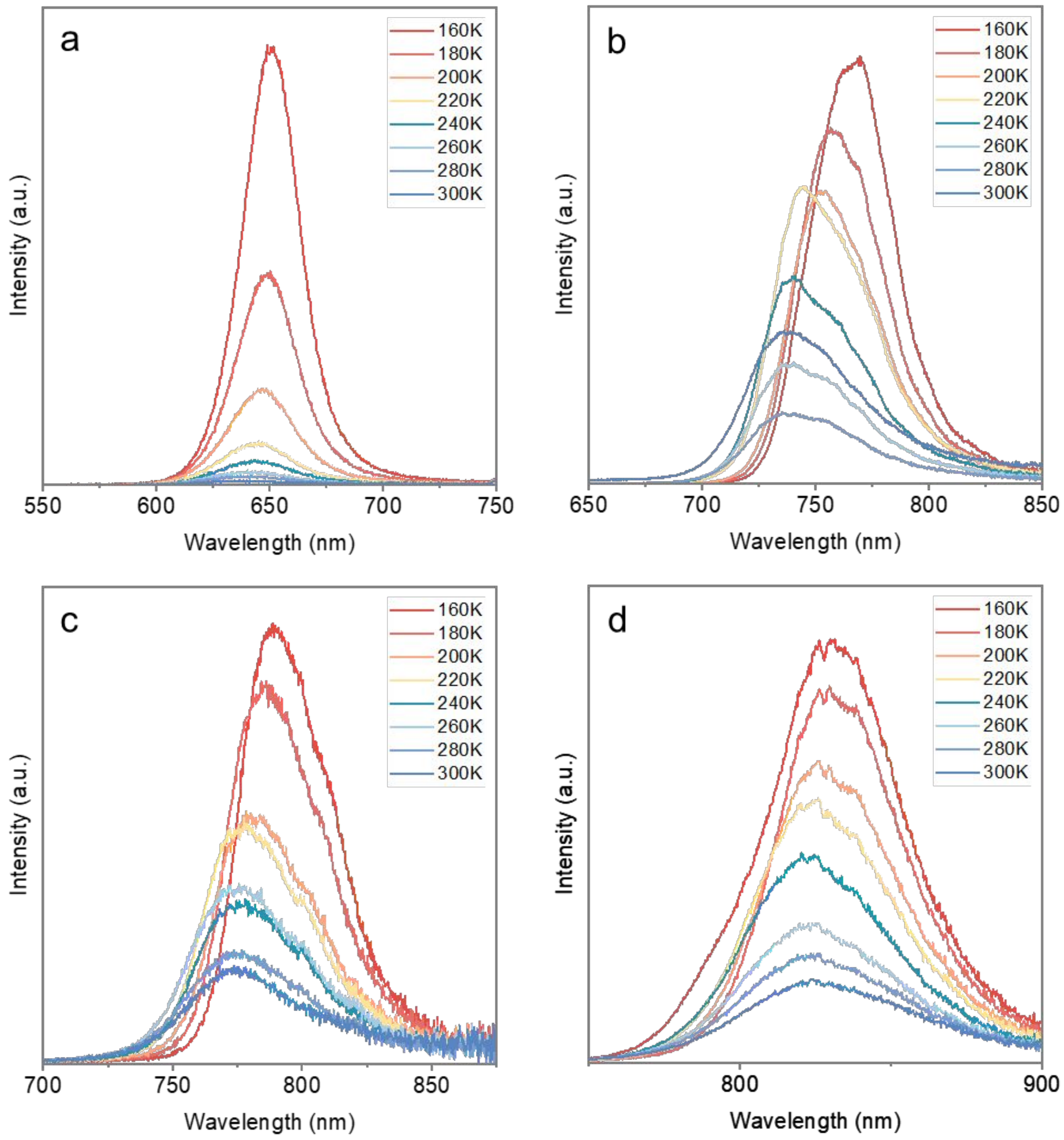

Figure S26. Temperature-dependent PL spectra. (a) $(5IPA3)_2SnI_4$ (n = 1), **(b)** $(5IPA3)_2(MA)Sn_2I_7$ (n = 2), **(c)** $(5IPA3)_2(MA)_2Sn_3I_{10}$ (n = 3), and **(d)** $(5IPA3)_2(MA)_3Sn_4I_{13}$ (n = 4) perovskite nanowires, respectively.

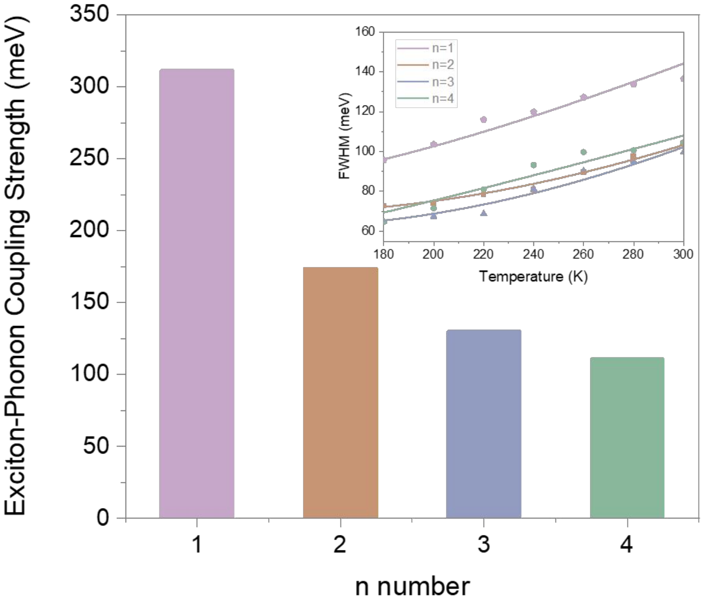

Figure S27. Extraction of exciton-phonon coupling strength using $(5IPA3)_2SnI_4$ (n = 1), $(5IPA3)_2(MA)Sn_2I_7$ (n = 2), $(5IPA3)_2(MA)_2Sn_3I_{10}$ (n = 3), and $(5IPA3)_2(MA)_3Sn_4I_{13}$ (n = 4) by fitting the full width at half maximum (FWHM) versus temperature. The fitting of the temperature-dependent PL broadening was performed using

$$\Gamma(T)=\Gamma_0+\frac{\Gamma_{LO}}{e^{E_{LO}/K_B T}-1} ,$$

Where Γ_0 is inhomogeneous broadening, Γ_{LO} is the coupling strength of the exciton-phonon interaction, E_{LO} is an energy of the LO phonon, T is the temperature, and k_B is the Boltzmann constant. Data from 180 K to 300 K were fitted to extract the exciton–LO phonon coupling strength.

Table S4. Fitting parameters of exciton-photon coupling in $(5IPA3)_2(MA)_{n-1}Sn_nI_{3n+1}$ (n = 1 - 4) nanowires.

Materials	Γ_0 (meV)	Γ_{LO} (meV)	E_{LO} (meV)	R^2
$(5IPA3)_2SnI_4$	78.0 ± 4	311.1 ± 26	45.0 ± 1	0.98
$(5IPA3)_2(MA)Sn_2I_7$	67.9 ± 3	173.6 ± 13	35.2 ± 1	0.97
$(5IPA3)_2(MA)_2Sn_3I_{10}$	55.6 ± 4	129.8 ±14	33.4 ± 1	0.96
$(5IPA3)_2(MA)_3Sn_4I_{13}$	45.2 ± 5	110.6 ± 19	31.8 ± 1	0.98

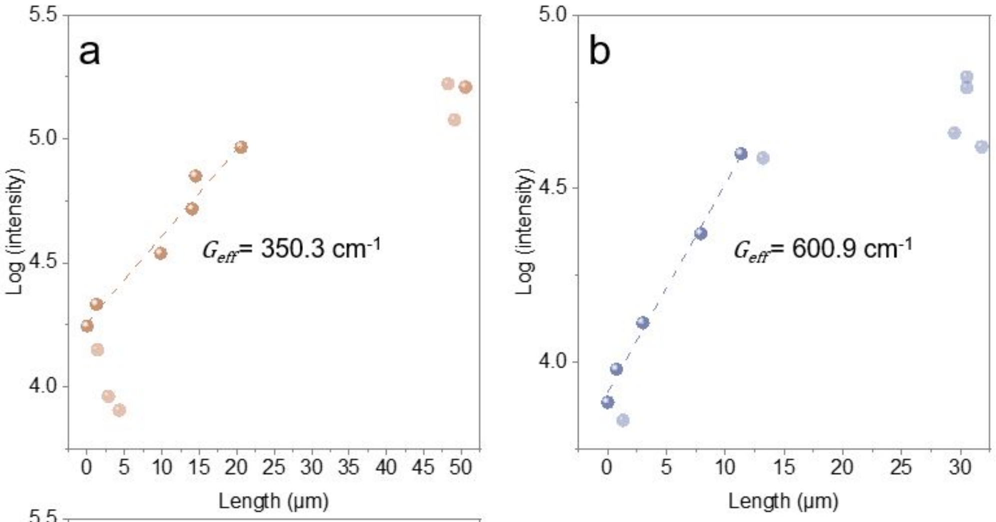

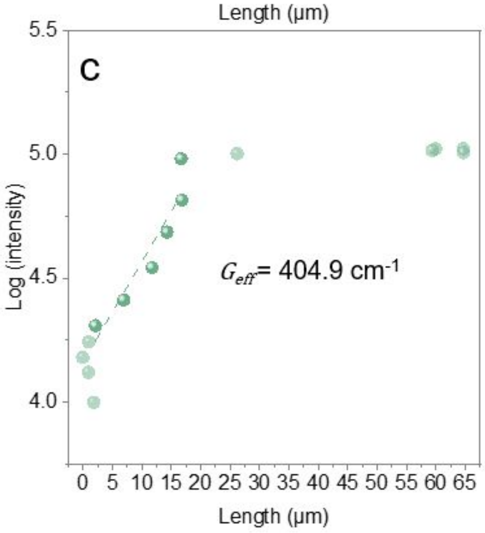

d

	n2	n3	n4
Sample size	6	6	3
Mean gain (cm^{-1})	249.8	246.4	232.8
std error (cm^{-1})	74.5	92.4	94.5
Max gain (cm^{-1})	350.3	600.9	404.9

Figure S28. Measurements of effective gain for individual **(a)** n = 2, **(b)** n = 3, and **(c)** n = 4, nanowires respectively, determined with the variable stripe length (VSL) method. The wire length was normalized to account for the uniformity of the beam. The gain was determined from the slope of a linear regression model of the log(counts) vs normalized wire length. **(d)** effective gain statistics for n = 2, 3, and 4 nanowires.

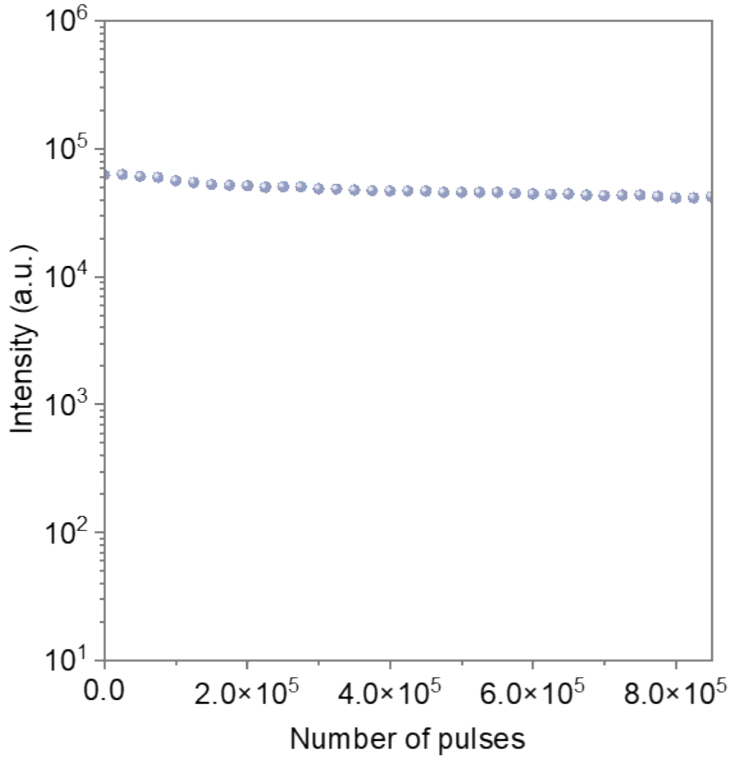

Figure S29. Additional integrated lasing intensity over pulses from a $(5IPA3)_2(MA)_2Sn_3I_{10}$ (n = 3) nanowire.

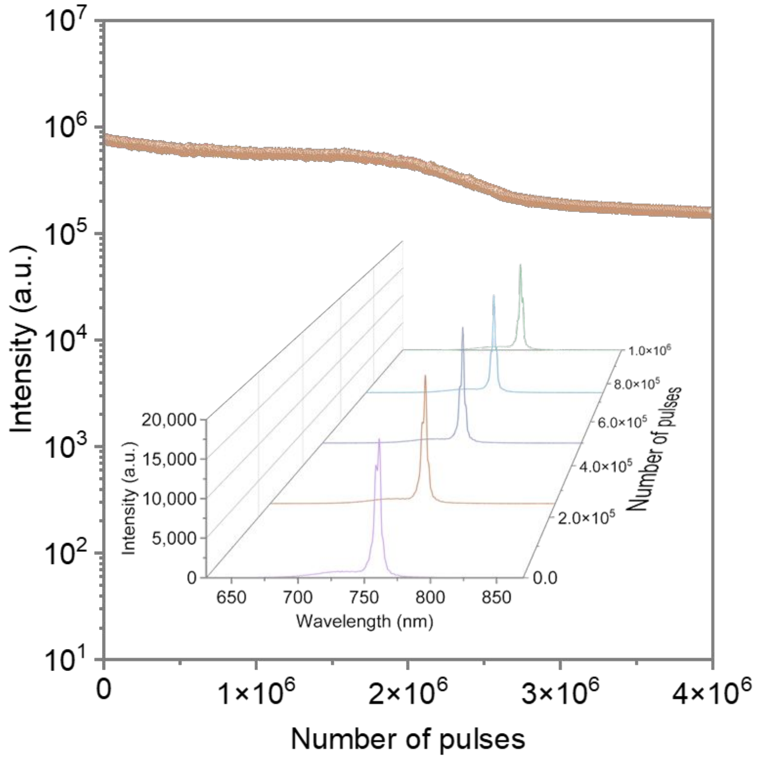

Figure S30. Integrated lasing intensity over pulses from a $(5IPA3)_2(MA)Sn_2I_7$ (n = 2) nanowire. Inset shows the spectral evolution of lasing emission (300 gr/mm grating) under continuous excitation at a repetition rate of 1 kHz at RT.

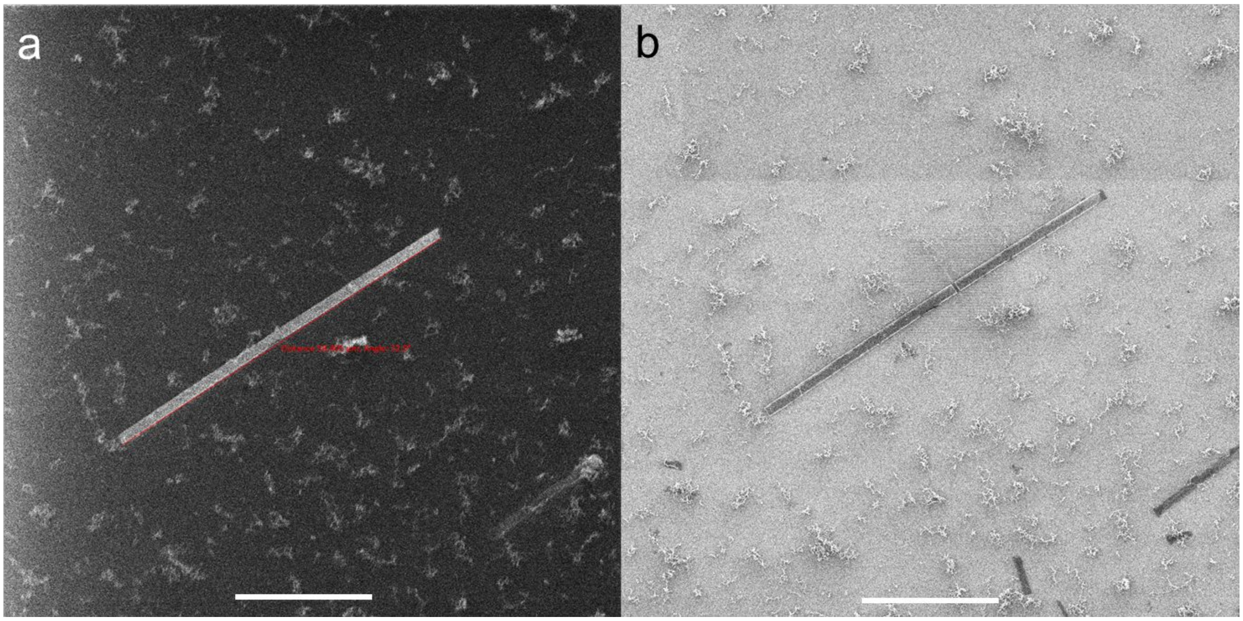

Figure S31. Helium ion microscopy images under Neon FIB milling. (a) Before cutting nanowire. **(b)** Axially coupled $(5IPA3)_2(MA)_2Sn_3I_{10}$ (n = 3) nanowire on Si/SiO_2 substrate after cutting using FIB. (scale bars: 20 µm)

Table S5. Comparison of length, threshold, and mode spacing, before and after neon FIB milling. The calculated mode spacing was obtained from the plot of mode spacing versus reciprocal wire length.

	Length (μm)	Threshold (μJ/cm^2)	Calculated Mode Spacing (meV)	Actual Mode Spacing (meV)
**Pre-modification**	58.4	1138	1.9	2.2
**Cleaved-coupled cavity**	25, 33.4	1178	3.4, 4.5	5.5

Note S1. Resonances in FP nanowire cavities

Multiple resonances

The nanowire cross-sectional geometry supports multiple transverse waveguide modes with varying effective refractive indices, n_{eff}, and edge-facet reflection coefficients (see simulation section below). Light propagating in such waveguide mode can result in FP resonances with different free spectral ranges (FSRs). The result is beating-like pattern in the PL spectrum. Above the lasing threshold, the prominence of each set of FP resonances strongly depends on the efficiency of reflection at the waveguide facets,[7] eliminating the consideration of many sets of resonances. The multiple waveguide mode explanation can account unexpected peak broadening resulting from the overlap of two FP resonances with similar FSRs.

Corrected calculations of n_{eff}

To illustrate the different sets of FP modes, we analyze the FSR and calculate the refractive index calculated from the lasing spectra of a $n = 3$ wire (Figure S32) using the expression for FSR $\Delta v = \frac{hc}{2\, n_{eff}\, L}$, where h is plancks constant, c is the speed of light, and L is the length of the nanowire. First, we analyze n_{eff} when considering all of the lasing peaks showing that the value is unrealistic. We then assigned the peaks to one of two sets of FP resonances and compute n_{eff} for each set (Table S6), arriving at values similar to literature.[8-9]

An alternative method to calculate mode spacing is by examining the slope of the spacing vs. 1/L plot (Figure 3e) with respect to the FSR relation fitted with a linear regression model with the y-intercept fixed at 0 and two outliers removed from the $n = 3$ data. An integer divisor of two was introduced to account for the approximate number of dominant sets of FP resonances. The result is shown in Table S7 wand have similar refractive indices as the previous calculations.

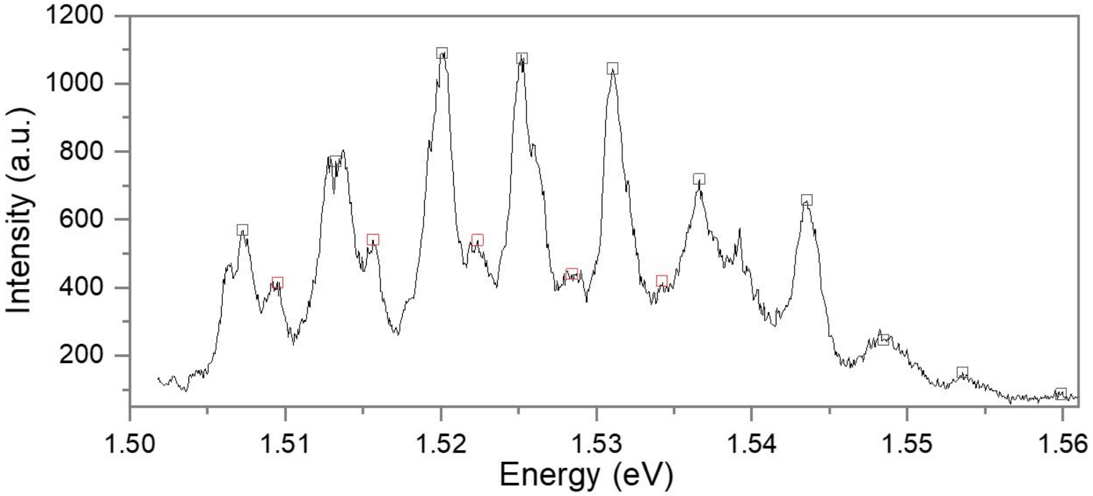

Figure S32. Nanolasing spectrum of an n = 3 nanowire for mode spacing analysis. The plot includes markers of the potential peaks associated with two potential sets of FP resonance mode (black and red boxes) and used for FSR calculations. Not all peaks are intended to be marked because they were not associated with the main set of FP resonances identified.

Table S6. FSR and n_{eff} calculations when considering various peaks

	ΔV_{avg}	SE	n_{eff}
All Peaks	3.757	0.360	3.49
Mode 1	5.844	0.234	2.24
Mode 2	6.173	0.175	2.12

Table S7. Calculations of n_{eff} from slope of the mode spacing vs reciprocal length data

n	Slope (meV μm)	n_{eff}
2	130.6	2.37
3	130.2	2.38
4	147	2.11

Multimode optical simulations

Lumerical's S-parameter feature, which combines both a mode solver and FDTD simulation to determine the input/output coupling of waveguide modes, was applied to determine the field profile and respective reflectivity for each waveguide mode with a TE component of over 70%. Nanowire geometries from several *n* = 3 perovskite nanowires were measured from experimental data (Table S6) and a refractive index of 2.5 was used in accordance with reference.[9] Notably, the waveguide modes associated with the highest reflectivities can include higher order modes (Figure

S33), indicating modes with a wide variety of effective refractive indices (n_{eff}) contributed to lasing (Table S9). Further, a smaller nanowire cross section is associated with significantly lower reflecitivites (R < 30%) than the reflectivity of larger cross section wires (R > 70%), potentially having a significant effect on lasing performance (Table S8).

Table S8. Dimension of simulated nanowire cross sections and summary information

Wire	Width (um)	Height (um)	n_{eff} of highest reflectivity mode	R of highest reflectivity mode
1	1.4	0.15	1.617	0.286
2	2.41	0.366	1.837	0.718
3	3.04	0.122	1.558	0.378
4	3.2	0.258	1.738	0.846
5	0.69	0.148	1.634	0.650

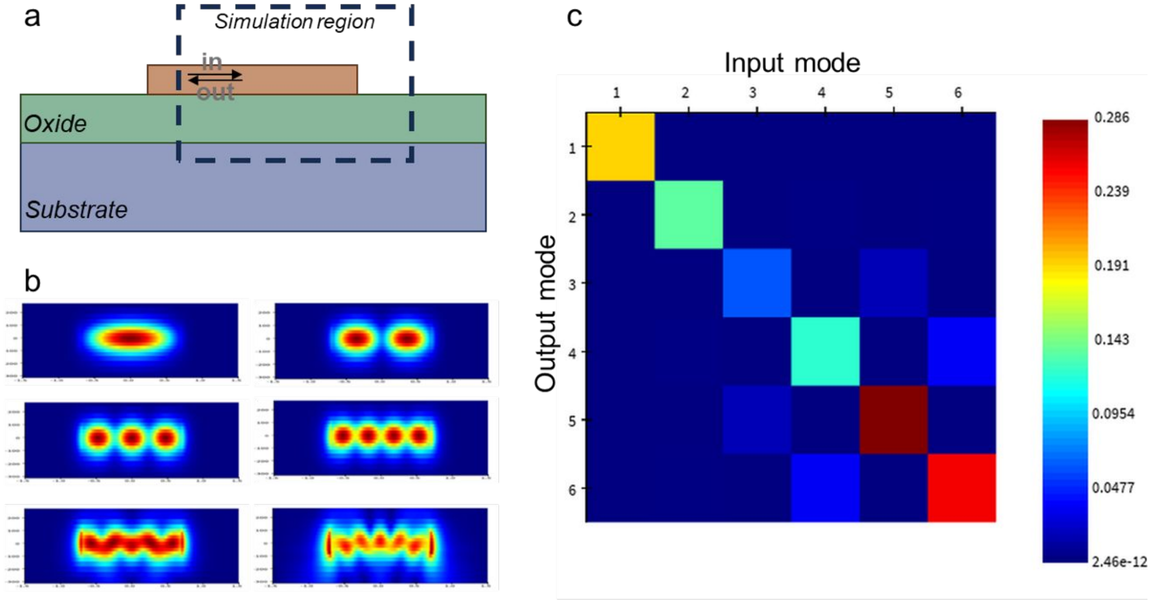

Figure S33. Simulation of modes in nanowire. a. Cross sectional view of nanowire indicating the simulation region and placement of input and output ports for S-parameter analysis. **b**. Calculated TE mode profiles for Wire 1 in Table S3. **c**. FDTD simulation of $|S|^2$ representing the overlap of input and output modes after reflection. The diagonal represents the reflection coefficients of each mode at the edge facet of the nanowire.

Table S9. Properties of simulated waveguide modes

Wire 1			Wire 2			Wire 3			Wire 4			Wire 5		
n_{eff}	R	TE	n_{eff}	R	TE	n_{eff}	R	TE	n_{eff}	R	TE	n_{eff}	R	TE
2.118	0.191	1.000	2.456	0.309	1.000	2.022	0.162	1.000	2.365	0.295	1.000	2.316	0.263	1.000
2.061	0.134	0.999	2.440	0.288	1.000	2.009	0.150	1.000	2.355	0.281	1.000	2.265	0.194	1.000
1.963	0.060	0.997	2.412	0.251	1.000	1.988	0.130	1.000	2.339	0.257	1.000	2.176	0.096	0.999
1.819	0.120	0.991	2.372	0.197	1.000	1.958	0.105	1.000	2.315	0.222	1.000	2.047	0.131	0.995
1.617	0.286	0.867	2.320	0.137	0.999	1.918	0.078	0.999	2.285	0.179	1.000	1.872	0.448	0.834
1.358	0.254	0.770	2.255	0.122	0.996	1.869	0.051	0.999	2.248	0.130	1.000	1.634	0.650	0.952
			2.176	0.253	0.998	1.809	0.029	0.998	2.203	0.084	0.998	1.236	0.007	0.701
			2.081	0.475	0.997	1.738	0.051	0.996	2.150	0.059	0.999			
			2.018	0.336	0.994	1.655	0.219	0.990	2.088	0.134	0.909			
			1.998	0.296	0.997	1.558	0.378	0.864	2.017	0.366	0.997			
			1.969	0.610	0.994	1.445	0.358	0.786	1.936	0.597	0.996			
			1.963	0.231	0.994	1.304	0.114	0.739	1.843	0.778	0.993			
			1.912	0.182	0.881	0.859	0.005	0.687	1.738	0.846	0.987			
			1.851	0.105	0.949				1.629	0.118	0.999			
			1.837	0.718	0.981				1.618	0.768	0.974			
			1.767	0.132	0.965				1.614	0.126	0.991			
			1.685	0.680	0.783				1.590	0.093	0.995			
			1.663	0.316	0.958				1.553	0.072	0.907			
			1.493	0.676	0.791				1.512	0.052	0.973			
			1.382	0.338	0.746				1.483	0.607	0.825			
									1.454	0.031	0.924			

Note S2. NMR spectra

Starting material, dimethyl 5-hydroxyisophthalate

^{1}H NMR (400 MHz, $CDCl_3$)

δ 8.26 (s, Ph-H, 1H), 7.72 (s, Ph-H, 2H), 3.92 (s, -O-CH_3, 6H).

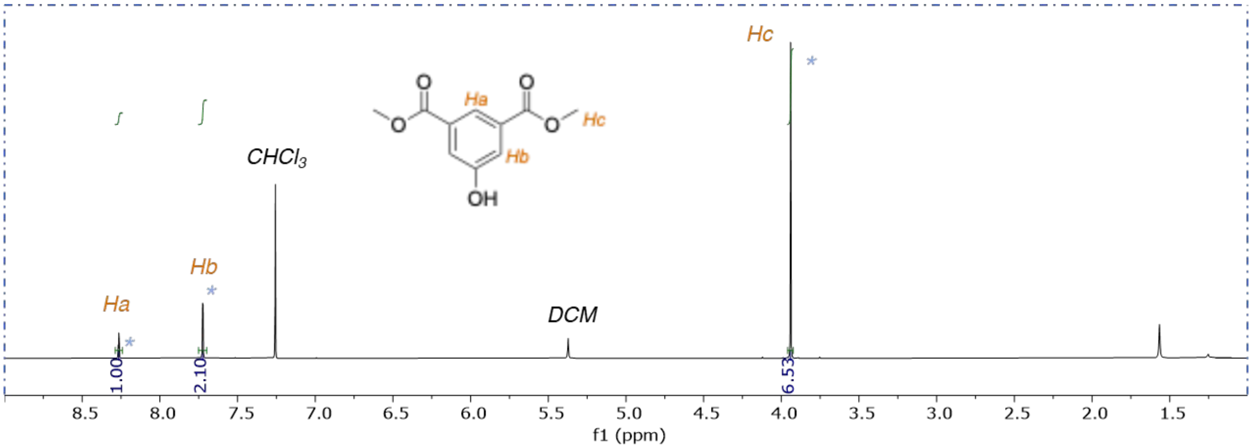

5IPA3-Boc, dimethyl 5-(2-((tert-butoxycarbonyl)amino)ethoxy)isophthalate

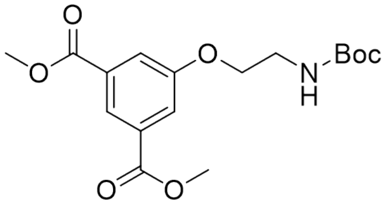

5IPA3-Boc

^{1}H NMR (400 MHz, $CDCl_3$)

δ 8.27 (s, Ph-H, 1H), 7.74 (s, Ph-H, 2H), 4.99 (s, -NH, 1H), 4.11 (m, O-CH_2-, 2H), 3.83 (s, -O-CH_3, 6H), 3.25 (m, CH_2-NH, 2H), 1.46 (s, -Boc, 9H).

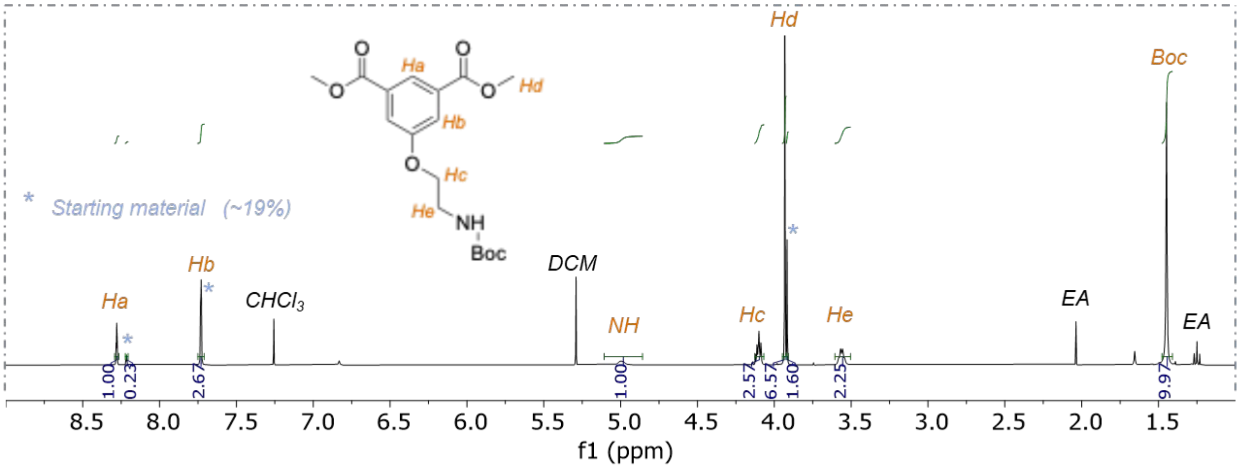

5IPA3-I, 2-(3-carboxy-5-(methoxycarbonyl)phenoxy)ethan-1-aminium iodide

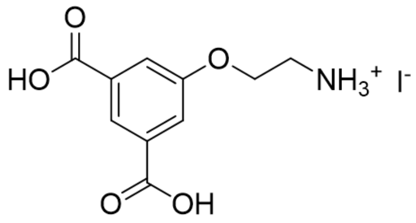

5IPA3-I

^{1}H NMR (400 MHz, DMSO-d6)

δ 13.3 (s, -COOH, 2H), 8.11 (s, Ph-H, 1H), 7.97 (s, $-NH_3$, 2H), 7.71 (s, Ph-H, 2H), 4.28 (m, O-CH2-, 2H), 3.25 (m, CH_2-NH_3, 2H).

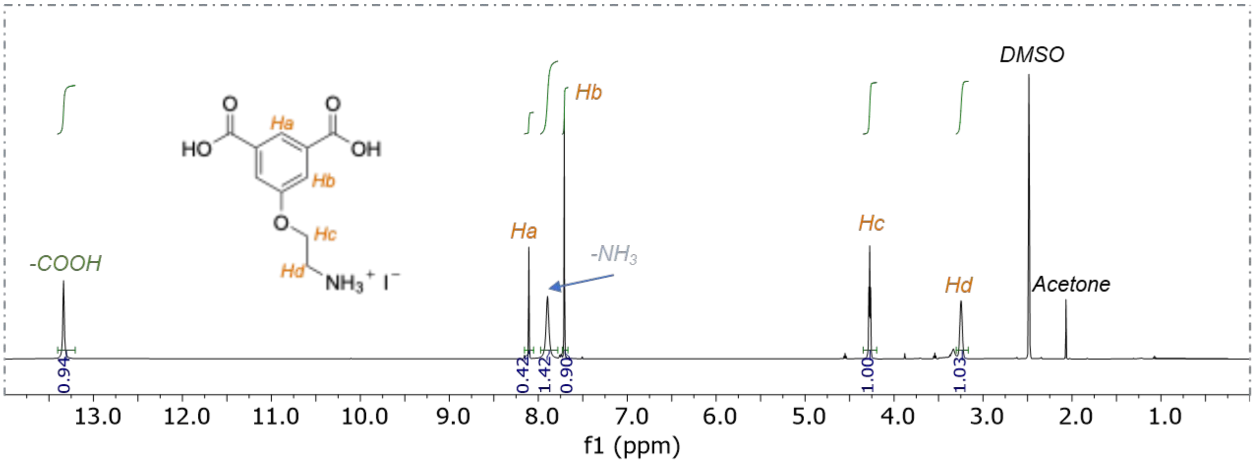

^{13}C NMR (126 MHz, DMSO-d6)

δ 166.6, 158.4, 133.3, 123.2, 119.9, 65.4, 38.6.

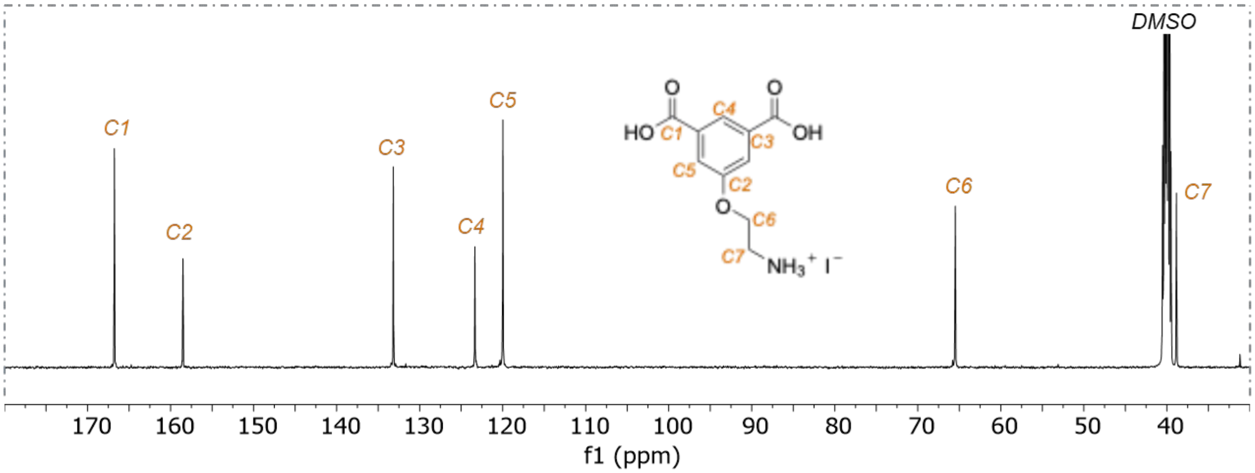

5IPA3-Br, 2-(3-carboxy-5-(methoxycarbonyl)phenoxy)ethan-1-aminium bromide

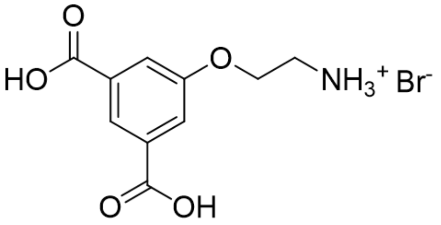

5IPA3-Br

^{1}H NMR (400 MHz, DMSO-d6)

δ 13.3 (s, -COOH, 2H), 8.12 (s, Ph-H, 1H), 7.98 (s, $-NH_3$, 2H), 7.7 (s, Ph-H, 2H), 4.28 (m, O-CH_2-, 2H), 3.25 (m, CH_2-NH_3, 2H).

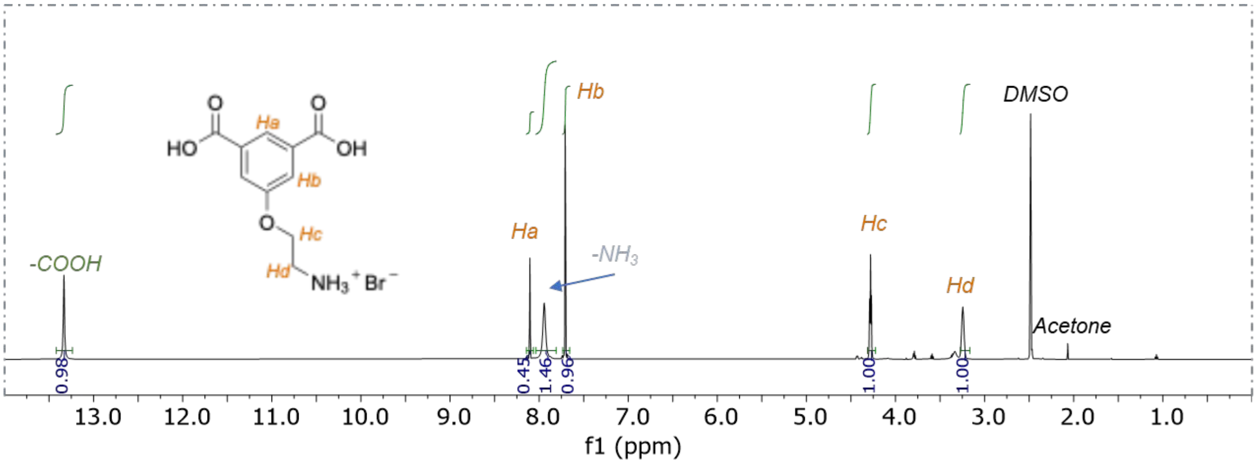

^{13}C NMR (126 MHz, DMSO-d6)

δ 166.6, 158.4, 133.3, 123.2, 119.8, 65.2, 38.6.

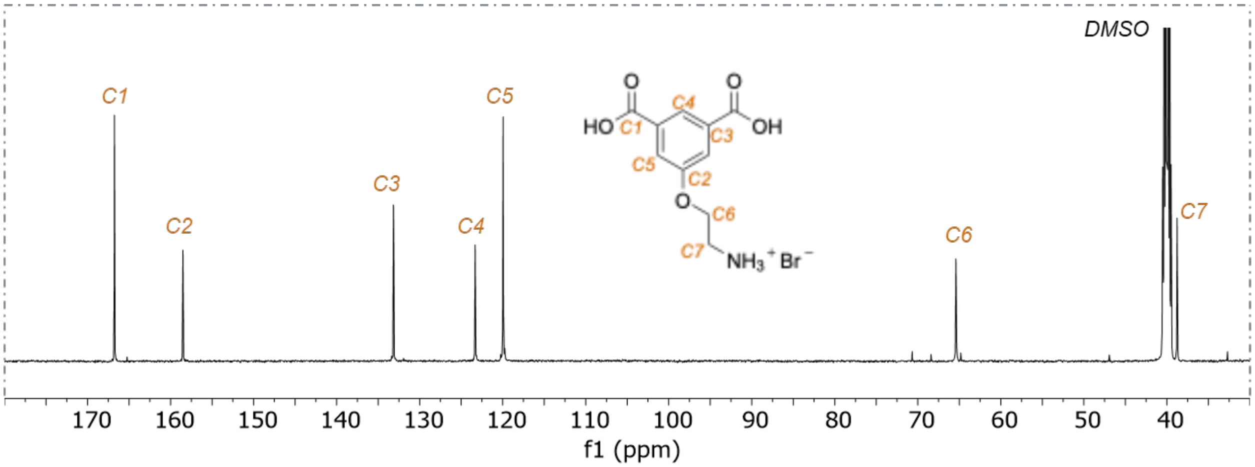

Note S3. Crystallography data

Table S10. Crystal data and structure refinement for $(5IPA3)_2PbBr_4$

CCDC #	2470258
Moiety formula	$C_{20}H_{24}Br_4N_2O_{10}Pb$
Formula weight	979.24
Temperature [K]	150(2)
Crystal system	orthorhombic
Space group (number)	$Pnma$ (62)
a [Å]	7.6248(17)
b [Å]	44.413(17)
c [Å]	8.569(3)
α [°]	90
β [°]	90
γ [°]	90
Volume [Å^3]	2901.6(16)
Z	4
ρ_{calc} [gcm^{-3}]	2.242
μ [mm^{-1}]	11.374
F(000)	1840
Crystal size [mm^3]	0.040×0.050×0.550
Crystal colour	colourless
Crystal shape	needle
Radiation	MoK_α (λ=0.71073 Å)
2θ range [°]	4.84 to 61.01 (0.70 Å)
Index ranges	$-9 \leq h \leq 10$<br>$-56 \leq k \leq 63$<br>$-12 \leq l \leq 11$
Reflections collected	25048
Independent reflections	4467<br>R_{int} = 0.0433<br>R_{sigma} = 0.0263
Completeness to θ = 25.242°	99.3 %
Data / Restraints / Parameters	4467 / 0 / 178
Absorption correction T_{min}/T_{max} (method)	0.1019 / 0.2694<br>(multi-scan)
Goodness-of-fit on F^2	1.066
Final R indexes [$I \geq 2\sigma(I)$]	R_1 = 0.0212<br>wR_2 = 0.0412
Final R indexes [all data]	R_1 = 0.0274<br>wR_2 = 0.0427
Largest peak/hole [eÅ^{-3}]	1.19/−2.08

Table S11. Crystal data and structure refinement for intercalated $(5IPA3)_2SnI_4$

CCDC #	2470259
Moiety formula	$C_{44}H_{60}I_8N_4O_{26}Sn_2$
Formula weight	2313.54
Temperature [K]	150(2)
Crystal system	monoclinic
Space group (number)	$P2_1/n$ (14)
a [Å]	17.032(4)
b [Å]	8.7427(11)
c [Å]	46.503(13)
α [°]	90
β [°]	100.44(2)
γ [°]	90
Volume [Å^3]	6810(3)
Z	4
ρ_{calc} [gcm^{-3}]	2.257
μ [mm^{-1}]	34.972
F(000)	4336
Crystal size [mm^3]	0.010×0.040×0.230
Crystal colour	red
Crystal shape	plate
Radiation	CuK_α (λ=1.54178 Å)
2θ range [°]	5.28 to 160.57 (0.78 Å)
Index ranges	$-20 \leq h \leq 21$<br>$-11 \leq k \leq 11$<br>$-58 \leq l \leq 58$
Reflections collected	64780
Independent reflections	14508<br>R_{int} = 0.1293<br>R_{sigma} = 0.1450
Completeness to θ = 67.679°	99.9 %
Data / Restraints / Parameters	14508 / 16 / 788
Absorption correction T_{min}/T_{max} (method)	0.0380 / 0.1784<br>(multi-scan)
Goodness-of-fit on F^2	1.102
Final *R* indexes [$I \geq 2\sigma(I)$]	R_1 = 0.0796<br>wR_2 = 0.2022
Final *R* indexes [all data]	R_1 = 0.0973<br>wR_2 = 0.2146
Largest peak/hole [eÅ^{-3}]	2.77/−1.90

Refinement details

Twinning emulating the C-centered monoclinic symmetry was applied (two-fold rotation around 1 0 0 or around reciprocal (2 0 -1), TWIN law 1 0 0 0 -1 0 -1 0 -1). The twinning ratio refined to 0.656 to 0.344. Water H atom positions were initially refined and O-H and H...H distances were restrained to 0.84(2) and 1.36(2) Angstrom, respectively, while a damping factor was applied and were further restrained based on hydrogen bonding considerations. In the final refinement cycles, the H atoms were constrained to ride on their carrying oxygen atom (AFIX 3) and the damping factor was removed.

Table S12. Crystal data and structure refinement for intercalated $(5IPA3)_2(MA)Sn_2I_7$

CCDC #	2470260
Moiety formula	$C_{21}H_{30}I_7N_3O_{10}Sn_2$[+solvent]
Formula weight	1610.16
Temperature [K]	150(2)
Crystal system	orthorhombic
Space group (number)	$Pmc2_1$ (26)
a [Å]	28.502(5)
b [Å]	17.114(3)
c [Å]	8.921(3)
α [°]	90
β [°]	90
γ [°]	90
Volume [$Å^3$]	4351.5(18)
Z	4
ρ_{calc} [gcm^{-3}]	2.458
μ [mm^{-1}]	48.495
F(000)	2912
Crystal size [mm^3]	0.001×0.050×0.190
Crystal colour	black
Crystal shape	plate
Radiation	CuK_α (λ=1.54178 Å)
2θ range [°]	6.02 to 160.84 (0.78 Å)
Index ranges	$-36 \le h \le 36$<br>$-21 \le k \le 21$<br>$-11 \le l \le 8$
Reflections collected	51969
Independent reflections	8743<br>R_{int} = 0.1595<br>R_{sigma} = 0.1585
Completeness to θ = 67.679°	99.9 %
Data / Restraints / Parameters	8743 / 249 / 420
Absorption correction T_{min}/T_{max} (method)	0.4066 / 0.7543<br>(multi-scan)
Goodness-of-fit on F^2	1.067
Final R indexes [$I \geq 2\sigma(I)$]	R_1 = 0.0652<br>wR_2 = 0.1745
Final R indexes [all data]	R_1 = 0.1048<br>wR_2 = 0.2047
Largest peak/hole [$eÅ^{-3}$]	1.65/−2.26

Refinement details

Water H atom positions were initially refined and O-H and H...H distances were restrained to 0.84(2) and 1.36(2) Angstrom, respectively, while a damping factor was applied and were further restrained based on hydrogen bonding considerations. In the final refinement cycles, the H atoms were constrained to ride on their carrying oxygen atom (AFIX 3) and the damping factor was removed. The structure also exhibits large volume sections consisting of highly disordered solvate or other small molecules. No satisfactory model for the solvate molecules could be developed, and

the contribution of the solvate molecules was instead taken into account by reverse Fourier transform methods. First the LIST 8 function of Shelxl2019 was used and then the cif and fcf files were subjected to the SQUEEZE routine as implemented in the program Platon. The resultant files were used in the further refinement. A volume of 455 cubic Angstrom per unit cell containing 192 electrons was corrected for. As a result, the solvent molecules are not explicitly included in the structural model; therefore, a definitive moiety formula cannot not be reported.

Table S13: Crystal data and structure refinement for intercalated $(5IPA3)_2(MA)_2Sn_3I_{10}$

CCDC #	2470261
Moiety formula	$C_{94}H_{158}I_{40}N_{16}O_{47}Sn_{12}$
Formula weight	8764.63
Temperature [K]	150(2)
Crystal system	triclinic
Space group (number)	$P\bar{1}$ (2)
a [Å]	8.8903(18)
b [Å]	17.149(4)
c [Å]	35.392(7)
α [°]	97.039(10)
β [°]	97.218(13)
γ [°]	90.108(13)
Volume [Å^3]	5312.1(18)
Z	1
ρ_{calc} [gcm^{-3}]	2.740
μ [mm^{-1}]	57.168
F(000)	3930
Crystal size [mm^3]	0.001×0.050×0.190
Crystal colour	black
Crystal shape	flake
Radiation	CuK_α (λ=1.54178 Å)
2θ range [°]	5.07 to 143.58 (0.81 Å)
Index ranges	$-9 \leq h \leq 10$<br>$-20 \leq k \leq 20$<br>$-42 \leq l \leq 42$
Reflections collected	68711
Independent reflections	19595<br>R_{int} = 0.2036<br>R_{sigma} = 0.2281
Completeness to θ = 67.679°	99.4 %
Data / Restraints / Parameters	19595 / 2079 / 1068
Absorption correction T_{min}/T_{max} (method)	0.0432 / 0.1665<br>(multi-scan)
Goodness-of-fit on F^2	0.979
Final *R* indexes [$I \geq 2\sigma(I)$]	R_1 = 0.0976<br>wR_2 = 0.2600
Final *R* indexes [all data]	R_1 = 0.1514<br>wR_2 = 0.3066
Largest peak/hole [eÅ^{-3}]	3.35/−2.47

Refinement details

The structure closely emulates a double the volume C-centered cell with dimensions a = 70.223, b = 8.889, c = 17.149, beta = 97.11 and space group C2/c. Exact translational symmetry is broken by ordering of the iodine atoms of the central Sn-I layer (I1, I3, I6 and I7), which in C2/c are 1:1 disordered by two-fold axes. No disorder is observed in the triclinic structure once twinning is taken into account. All remaining Sn and I atoms as well as the larger cations obey the monoclinic C2/c setting methyl ammonium and acetic acid / water solvate molecules are too ill defined to tell).

Twinning emulating the C-centered monoclinic symmetry was applied (two-fold rotation around 1 0 0 or around reciprocal (2 0 -1), TWIN law 1 0 0 0 -1 0 -1 0 -1). The twinning ratio refined to 0.572(2) to 0.428(2). For the outer Sn-I layers, disorder over two moieties was refined for the iodine atoms. Minor moiety iodine atoms were constrained to have the same ADPs as their major moiety counterparts. Selected Sn-I bonds were restrained to be similar in length. No disorder was resolved for the methyl ammonium cations or the larger organic cations. Due to correlation by pseudosymmetry the four larger cations were restrained to have similar geometries, their benzene rings and adjacent atoms were restrained to be close to planar. A global similarity restraint for ADPs of C, N and O atoms was applied and U_{ij} components of ADPs of these atoms were restrained to be similar if closer to each other than 3 Angstrom (2 Angstrom for methyl ammonium, 8 Angstrom for acetic acid and water solvate molecules). Methyl ammonium C and N atoms were also restrained to be close to isotropic. Carboxylate H atoms were restrained to be coplanar with the carboxylate planes, and some were refined as 1:1 disordered, induced by disorder of acetic acid and water solvate molecules they are H-bonded to. Acetic acid and water solvate molecules form a close to planar layer between the other constituents parallel to the a-c-plane. Molecules are disordered by inversion centers, and generally ill-defined. They were refined as 1:1 disordered with three acetic acid and one water molecule in the asymmetric unit and approximately modelled by restraining the acetic acid molecules to expected geometries (using target values for bond distances and angles, planarity restraints) and intermolecular distances were restrained based on hydrogen bonding considerations. Water H atom positions were initially refined and O-H and H...H distances were restrained to 0.84(2) and 1.36(2) Angstrom, respectively, while a damping factor was applied and were further restrained based on hydrogen bonding considerations. In the final refinement cycles the H atoms were constrained to ride on their carrying oxygen atom (AFIX 3) and the damping factor was removed.

Table S14: Crystal data and structure refinement for intercalated $(5IPA3)_2(MA)_3Sn_4I_{13}$

CCDC #	2470265
Moiety formula	$C_{25}H_{48}I_{13}N_5O_{13}Sn_4$
Formula weight	2751.14
Temperature [K]	150(2)
Crystal system	orthorhombic
Space group (number)	$Pmc2_1$ (26)
a [Å]	41.084(4)
b [Å]	17.1512(17)
c [Å]	8.8447(11)
α [°]	90
β [°]	90
γ [°]	90
Volume [Å^3]	6232.4(11)
Z	4
ρ_{calc} [gcm^{-3}]	2.932
μ [mm^{-1}]	63.577
F(000)	4904
Crystal size [mm^3]	0.028×0.076×0.305
Crystal colour	black
Crystal shape	plate
Radiation	CuK_α (λ=1.54178 Å)
2θ range [°]	5.15 to 159.16 (0.78 Å)
Index ranges	$-51 \leq h \leq 52$<br>$-21 \leq k \leq 21$<br>$-8 \leq l \leq 11$
Reflections collected	58874
Independent reflections	11776<br>R_{int} = 0.0890<br>R_{sigma} = 0.1017
Completeness to θ = 67.679°	99.9 %
Data / Restraints / Parameters	11776 / 715 / 686
Absorption correction T_{min}/T_{max} (method)	0.0013 / 0.2267<br>(numerical)
Goodness-of-fit on F^2	1.054
Final R indexes [$I \geq 2\sigma(I)$]	R_1 = 0.0529<br>wR_2 = 0.1573
Final R indexes [all data]	R_1 = 0.0617<br>wR_2 = 0.1679
Largest peak/hole [eÅ^{-3}]	3.54/−2.19

Refinement details

The structure exhibits pseudo-inversion symmetry emulating Pbam symmetry. Inversion symmetry is broken by ordering of the iodine atoms of the two central Sn-I layers. In the Pbam setting they are 1:1 disordered by symmetry. In the Pmc2(1) setting the disorder is 92.5 to 7.5%. The nearby methyl ammonium ions are also ordered in the Pmc2(1) setting (no disorder resolved). The remainder of the structure is obeying centrosymmetric symmetry and atom parameters are thus correlated. The acetic acid / water layer is disordered by apparent inversion (by the inversion

centers of the Pbam setting), with close to 1:1 disorder. The disorder extends to the carboxylic acid H atoms to the organic ligands. Note: acetic acid and water molecules and the central methyl ammonium ions are located on crystallographic mirror planes. U_{ij} components of ADPs for disordered iodine atoms closer to each other than 2.0 Angstrom were restrained to be similar. Subject to these conditions the occupancy ratio refined to 0.925(3) to 0.075(3). The four methyl ammonium cations were restrained to have similar C-N bond lengths. U_{ij} components of ADPs for atoms closer to each other than 2.0 Angstrom or related by pseudo-inversion symmetry were restrained to be similar. Methyl and ammonium H atoms were initially allowed to rotate while a damping factor was applied. In the final refinement cycles the H atoms were constrained to ride on their carrying oxygen atom (AFIX 3) and the damping factor was removed. A rigid bond restraint (RIGU) was applied for the two ligands. U_{ij} components of ADPs for atoms related by pseudo-inversion symmetry were restrained to be similar. Carboxylate H atoms were refined as disordered following the pseudo-inversion disorder of the acetic acid-water layer. Most carboxylate H atoms were restrained to be coplanar with their COO group. Acetic acid C-C bonds were restrained to a target value of 1.53(2) Angstrom. C-O bond lengths and C-C-O bond angles were restrained to be similar for all acetic acid molecules. Water H atom positions were initially refined and O-H and H...H distances were restrained to 0.84(2) and 1.36(2) Angstrom, respectively, while a damping factor was applied. Some water H atom positions were further restrained based on hydrogen bonding considerations. In the final refinement cycles the H atoms were constrained to ride on their carrying oxygen atom (AFIX 3) and the damping factor was removed. Subject to these conditions the acetic acid-water occupancy ratio refined to 0.530(17) to 0.470(17). Refined as a 2-component inversion twin.

References

(1) Liang, Y.; Shang, Q.; Wei, Q.; Zhao, L.; Liu, Z.; Shi, J.; Zhong, Y.; Chen, J.; Gao, Y.; Li, M.; Liu, X.; Xing, G.; Zhang, Q., Lasing from Mechanically Exfoliated 2D Homologous Ruddlesden–Popper Perovskite Engineered by Inorganic Layer Thickness. *Adv. Mater.* **2019,** *31* (39), 1903030. Doi: 10.1002/adma.201903030

(2) Gao, W.; Wei, Q.; Wang, T.; Xu, J.; Zhuang, L.; Li, M.; Yao, K.; Yu, S. F., Two-Photon Lasing from Two-Dimensional Homologous Ruddlesden–Popper Perovskite with Giant Nonlinear Absorption and Natural Microcavities. *ACS Nano* **2022,** *16* (8), 13082-13091. Doi: 10.1021/acsnano.2c05726

(3) Park, J. Y.; Song, R.; Liang, J.; Jin, L.; Wang, K.; Li, S.; Shi, E.; Gao, Y.; Zeller, M.; Teat, S. J.; Guo, P.; Huang, L.; Zhao, Y. S.; Blum, V.; Dou, L., Thickness control of organic semiconductor-incorporated perovskites. *Nat. Chem.* **2023,** *15* (12), 1745-1753. Doi: 10.1038/s41557-023-01311-0

(4) Li, Y.; Zhou, H.; Xia, M.; Shen, H.; Wang, T.; Gao, H.; Sheng, X.; Han, Y.; Chen, Z.; Dou, L.; Zhu, H.; Shi, E., Phase-pure 2D tin halide perovskite thin flakes for stable lasing. *Sci. Adv.* **2023,** *9* (32), eadh0517. Doi: 10.1126/sciadv.adh0517

(5) Shao, W.; Kim, J. H.; Simon, J.; Nian, Z.; Baek, S.-D.; Lu, Y.; Fruhling, C. B.; Yang, H.; Wang, K.; Park, J. Y.; Huang, L.; Yu, Y.; Boltasseva, A.; Savoie, B. M.; Shalaev, V. M.; Dou, L., Molecular templating of layered halide perovskite nanowires. *Science* **2024,** *384* (6699), 1000-1006. Doi: 10.1126/science.adl0920

(6) Zhang, M.; Jin, L.; Zhang, T.; Jiang, X.; Li, M.; Guan, Y.; Fu, Y., Two-dimensional organic-inorganic hybrid perovskite quantum-well nanowires enabled by directional noncovalent intermolecular interactions. *Nat. Commun.* **2025,** *16* (1), 2997. Doi: 10.1038/s41467-025-58166-x

(7) Maslov, A. V.; Ning, C. Z., Reflection of guided modes in a semiconductor nanowire laser. *Appl. Phys. Lett.* **2003,** *83* (6), 1237-1239. Doi: 10.1063/1.1599037

(8) Zhang, L.; Sun, C.; He, T.; Jiang, Y.; Wei, J.; Huang, Y.; Yuan, M., High-performance quasi-2D perovskite light-emitting diodes: from materials to devices. *Light Sci. Appl.* **2021,** *10* (1), 61. Doi: 10.1038/s41377-021-00501-0

(9) Alvarado-Leaños, A. L.; Cortecchia, D.; Saggau, C. N.; Martani, S.; Folpini, G.; Feltri, E.; Albaqami, M. D.; Ma, L.; Petrozza, A., Lasing in Two-Dimensional Tin Perovskites. *ACS Nano* **2022,** *16* (12), 20671-20679. Doi: 10.1021/acsnano.2c07705
